# Multimodal personalised executive function intervention (E-Fit) for school-aged children with complex congenital heart disease in Switzerland: a randomised controlled feasibility study

**DOI:** 10.1136/bmjopen-2025-107681

**Published:** 2026-05-08

**Authors:** Alenka S Schmid, Melanie Ehrler, Silvia A Bunge, Oliver Kretschmar, Markus A Landolt, Valentin Rousson, Ruth O’Gorman Tuura, Flavia M Wehrle, Beatrice Latal

**Affiliations:** 1 Child Development Center, University Children’s Hospital Zurich, Zürich, Switzerland; 2 Children’s Research Center, University Children’s Hospital Zurich, Zürich, Switzerland; 3 Department of Forensic and Neurodevelopmental Sciences, King’s College London Institute of Psychiatry Psychology & Neuroscience, London, UK; 4 Department of Psychology & Helen Wills Neuroscience Institute, University of California Berkeley, Berkeley, California, USA; 5 Department of Cardiology, University Children’s Hospital Zurich, Zurich, Switzerland; 6 University of Zurich, Zürich, Switzerland; 7 Department of Psychosomatics and Psychiatry, University Children’s Hospital Zurich, Zürich, Switzerland; 8 Division of Child and Adolescent Health Psychology, Department of Psychology, University of Zurich, Zürich, Switzerland; 9 Division of Biostatistics Center for Primary Care and Public Health (Unisanté), University of Lausanne, Lausanne, Switzerland; 10 MR Research Centre, University Children’s Hospital Zurich, Zürich, Switzerland; 11 URPP Adaptive Brain Circuits in Development and Learning, University of Zurich, Zürich, Switzerland

**Keywords:** Paediatric cardiology, Feasibility Studies, Psychosocial Intervention, Congenital heart disease

## Abstract

**Objective:**

To explore the feasibility of a novel multimodal executive function intervention in school-aged children with complex congenital heart disease (cCHD).

**Design:**

Single-centre, single-blinded, randomised-controlled 8-week multimodal personalised executive function intervention (E-Fit) study. Outcomes were measured throughout the intervention, post-intervention (T1) and at 4-month follow-up (T2).

**Setting:**

Tertiary care centre between May 2022 and May 2024.

**Participants:**

Children 10 to 12 years (M=11.0, SD=0.9) with cCHD without a genetic diagnosis with infant open-heart surgery and reported difficulties (T-scores ≥60) on any of the summary scales of the parent- or teacher-reported Behavior Rating Inventory for Executive Function (BRIEF).

**Interventions:**

Children with cCHD were randomly assigned to one of two groups: the intervention or the control group. The 8-week intervention was multimodal including three modalities: (1) computerised executive function (EF) training 3×20 min/week with CogniFit; (2) a weekly, remote standardised 1:1 individual EF strategy coaching; (3) analogue games played at convenience. The control group completed activity logs.

**Feasibility measures:**

Acceptability: Acceptance and Feasibility Scale (AFS) and coach-rated engagement during coaching sessions. Demand: Number of completed computerised training, strategy coaching and analogue game sessions. Implementation: E-Fit Fidelity Measurement System, assessing adherence to core components. Practicality: Retention rate. Integration: AFS integration items. Exploratory efficacy: BRIEF, neuropsychological EF testing and psychosocial variables at baseline, post-intervention (8 weeks) and at 4-month follow-up.

**Results:**

We recruited 42 participants (N_female_=20). Acceptability: The intervention was acceptable, with moderate observed engagement. Demand: median number of computerised training sessions completed was 16 of 24 sessions (67%, (IQR; 6 to 19)), all children attended all scheduled coaching sessions, analogue games were played in total a median of 9 times (IQR 4 to 14). Implementation: Coaching sessions could be implemented by the coaches as intended. Practicality: Overall retention rate was 90%. Integration: E-Fit was well integrable into the home setting. Exploratory efficacy favoured the intervention group with improvements in the parent-rated Behavioral Regulation Index of the BRIEF (adjusted Hedge’s (g_A1_) = −0.408 to −0.903) and in social responsiveness (g_A1_ = −0.427 to −0.521) at T1 and at T2.

**Conclusions:**

E-Fit is a feasible intervention suggesting EF and social responsiveness improvements in children with cCHD. Motivational strategies to improve adherence to computerised training should be refined before a full-scale efficacy trial.

**Trial registration number:**

NCT05198583.

**Strengths and limitations of this study**

- Executive function intervention was developed collaboratively with input from patients and their families.
- Feasibility data were collected comprehensively, as recommended for feasibility studies.
- The multimodal nature of the intervention addresses the shortcomings of previous executive function intervention trials.
- Participants were unblinded following the baseline assessment, and some of the questionnaires were filled in in the presence of the investigators/coaches, potentially introducing a bias.
- Participants from low socioeconomic status backgrounds were under-represented due to recruitment difficulties.

## Introduction

 Congenital heart disease (CHD) is the most common birth defect with a prevalence of approximately 1%.^1^ Around one third of affected newborns have a complex congenital heart disease (cCHD) and require cardiopulmonary bypass surgery within the first year of life.^2^ With increased survival rates, the focus has shifted towards long-term neurodevelopmental morbidities, specifically executive dysfunction.^2^,^5^ Executive functions (EFs) are higher-level cognitive effortful control processes such as inhibition, cognitive flexibility and working memory that support complex behaviours such as reasoning, planning and decision-making.^6^,^10^ Children with cCHD face an elevated risk of difficulties across all these functions,^3 11^ which can impact mental^12^,^18^ and physical health^19^,^21^ and quality of life^22^,^24^ and contribute to academic^25^,^28^ and professional challenges.^29^

EF intervention studies for children with cCHD are, to date, limited to three studies, two of which involve the computerised Cogmed working memory training programme^30 31^ and one using LEGO-based therapy.^32^ The first study using Cogmed reported immediate postintervention improvements in performance-based working memory measures; however, these were not sustained longitudinally.^31^ The second study, also using Cogmed, found no improvement in working memory, although participants exhibited enhanced performance-based inhibitory control and parent-reported self-regulation at a 3-month follow-up.^30^ The third study using the LEGO-based therapy reported improvements in performance-based motor control, inhibitory control, risk selection and total EFs at immediate post-intervention, but had no follow-up assessment.^32^ Considering the limited benefits of existing EF interventions for patients with cCHD, it is essential to explore EF interventions in other clinical cohorts. A recent meta-analysis indicated that acquiring new strategies of self-regulation were the most effective for children with neurodevelopmental disorders.^33^ In children with autism and in adolescents with epilepsy, online individualised coaching sessions focussing on problem-solving strategies combined with computerised training targeting multiple EF domains improved EF behaviours.^34 35^ Further, analogue games, such as board and card games targeting EFs, have improved EFs in children with attention deficit hyperactivity disorder. Their interactive nature may promote better self-monitoring in children.^36 37^ Combining gamified computerised training paradigms with analogue games targeting reasoning and processing speed showed improvement in reasoning and speed tasks among healthy children from low socioeconomic backgrounds.^38^ Within these multimodal interventions, also multiple EF domains could be targeted. These interventions enabled targeting multiple EF domains across modalities. Given that children with cCHD are at a higher risk to face challenges across multiple EF domains, a multimodal intervention approach appears particularly beneficial for enhancing their EFs.

Importantly, identifying and addressing factors that influence the efficacy of an intervention within the target population is critical.^33^ Hence, the present study was informed by a comprehensive needs assessment to incorporate the perspectives of patients, their families, teachers and healthcare professionals in the development of the executive function intervention (E-Fit).^39^ In sum, E-Fit is a multimodal intervention including gamified computerised training, 1:1 coaching sessions and analogue games targeting a broad spectrum of EF domains. E-Fit is adaptive to the individual needs of children with cCHD and their families, aiming to maintain high motivation and commitment to the intervention.^40 41^ In this study, we aim to test the feasibility of E-Fit for 10-to-12-year-old children with cCHD and EF impairments.

## Methods

### Study design and population

**Figure 1:**
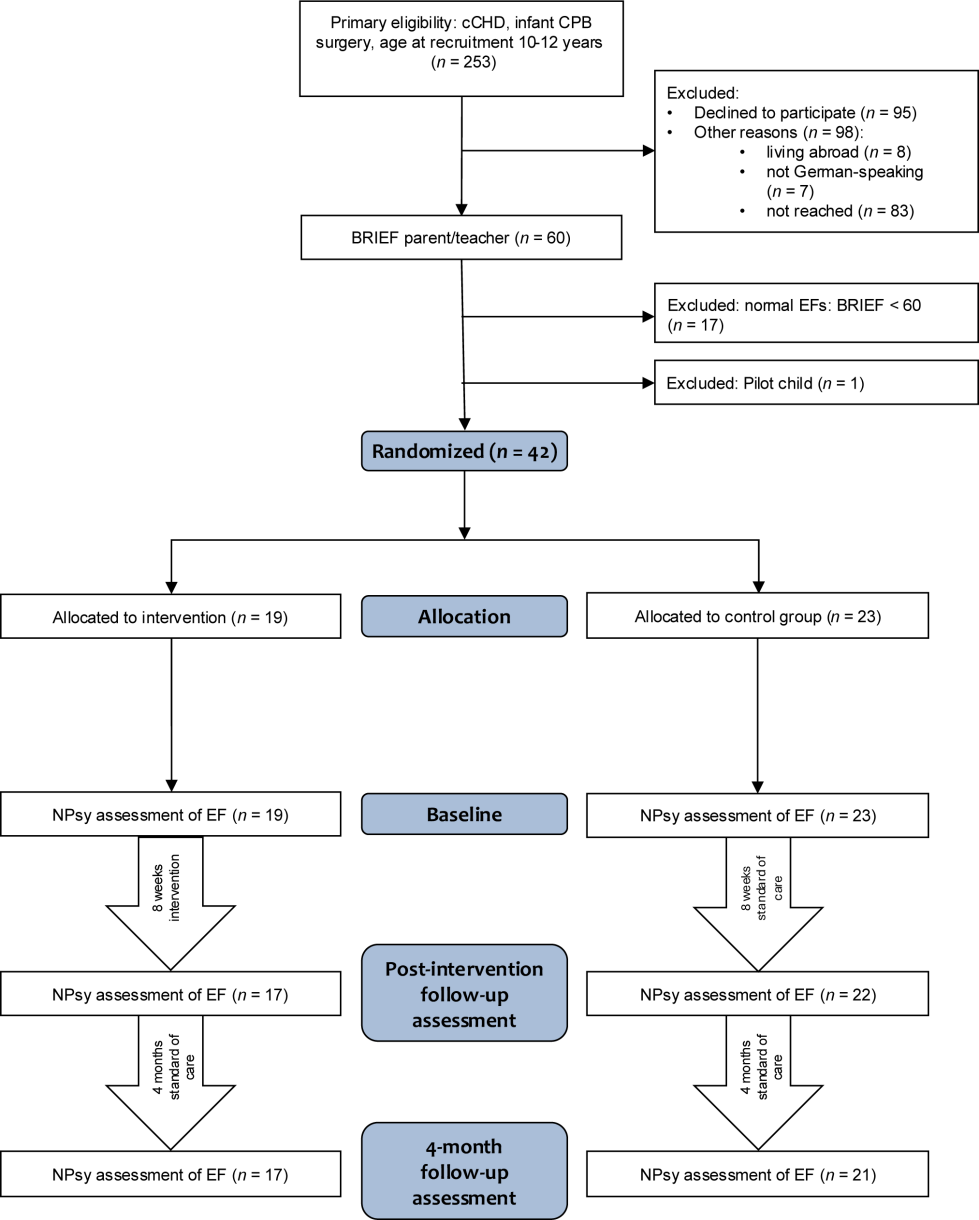
Study recruitment flowchart showing the number of participants identified, screened for eligibility, excluded, enrolled, and allocated to study groups. BRIEF, Behaviour Regulation Inventory of Executive Functions; cCHD, complex congenital heart disease; CPB, cardiopulmonary bypass; EF, executive functions; NPsy, neuropsychological.

This is a single-centre, single-blinded, randomised controlled study. Participants were recruited from May 2022 to November 2023. Primary eligibility criteria were children with cCHD who had undergone cardiopulmonary bypass surgery (CPB) within their first year of life at the University Children’s Hospital Zurich and who were 10 to 12 years of age at the time of recruitment. Exclusion criteria were diagnoses of a genetic comorbidity or dysmorphic syndrome or large cerebral lesions or injuries. See figure 1 for the flowchart.

Parents of primarily eligible patients were contacted by postal mail and phone. If families agreed to participate, parents and teachers filled in the Behavior Rating Inventory for Executive Function (BRIEF^42^) to rate the children’s everyday EFs. If the T-value on any summary scale of the BRIEF was ≥60, reflecting a borderline or clinically relevant EF problem, children were enrolled in the study and invited to a neuropsychological baseline assessment of EFs and intelligence quotient (IQ) lasting about 2.5 hours. The baseline assessments were performed by a psychologist (or psychologist in training) at the University Children’s Hospital Zurich. Afterwards, the participants were randomly assigned to either the E-Fit or control group using block randomisation (block size=4) to maintain group balance. Randomisation was stratified by maternal education. Subsequently, participants entered an 8-week intervention phase during which the intervention group engaged in E-Fit and regularly responded to questions assessing its feasibility. Patients in the control group completed an activity log by selecting activities from a list including school, leisure, physical activities and custom entries. This allowed us to monitor whether the control group independently engaged in EF-fostering activities included in E-Fit, and thereby assessing potential contamination of the control condition. Neuropsychological and psychosocial variables were collected from both groups at baseline, post-intervention (T1) and at a 4-month follow-up (T2). The 4-month follow-up was selected to be consistent with previous studies examining longer-term effects, in which such effects were variably observed.^30 31^

This study was registered on Clinical Trials.gov (NCT05198583) and approved by the ethics committee of the Canton of Zurich, Switzerland (Project ID 2021-02413). Written informed consent was obtained prior to participation from the participants’ legal guardians. The trial was conducted and reported according to the Standard Protocol Items: Recommendations for Interventional Trials (SPIRIT) guidelines.

### Intervention

#### E-Fit

The intervention lasted for 8 weeks.^39^ E-Fit targets various EF domains through a multimodal personalised approach consisting of three components tailored to the specific difficulties and needs of each child.

##### Computerised training

Gamified computerised training using the CogniFit Inc 2022 (CogniFit) platform, featuring 11 adaptive games for specific EF domains. For example, inhibition is trained using a frog jump challenge or a candy sorting task training planning skills. The training is intended to be performed on a computer, smartphone or tablet; the study team provides a device if necessary. The predefined routine for the computerised training involves three 20-minute sessions per week.

##### Strategy coaching

Weekly 60-minute remote standardised 1:1 coaching sessions with a psychologist were conducted remotely over StarLeaf (meet.starleaf.com^43^) or a secured Zoom session (uzh.zoom.ch^44^). The coaching targets strategies to overcome EF challenges in daily life and uses components from a published workbook.^45^ The strategy coaching comprises eight sessions and each session is structured and standardised. The first session primarily serves as a feedback session, during which the child and parents are informed about the results of the EF assessment. As noted by Pletschko *et al*,^46^ feedback of this kind already constitutes an element of neuropsychological intervention. Accordingly, the manual for the first session has been previously published and is taken from the book ‘Neuropsychologische Therapie mit Kindern und Jugendlichen’.^46^

For the subsequent sessions, 11 potential EF topics are available, each linked to a task from the workbook Smart but Scattered by Dawson and Guare.^45^ To identify the most relevant topics for each child, parents complete the corresponding questionnaire derived from the workbook. The six topics rated as most relevant are subsequently addressed in six strategy coaching sessions. Each of these sessions begins with a review of the previous week, followed by a brief story from Liebers *et al*,^47^,^50^ and concludes with completion of the selected task from the workbook. Which story and which part of it specifically is tailored to the respective topic of the session. For example, in one session, children would hear about a little dragon who is scared of a maths test. He cannot remember how to calculate the day before the test. But his friend the rabbit comes along and together they sing a song and breathe deeply. As the dragon calms down, he can remember everything and solve the last, preparing homework. Afterwards, the child is asked to reflect in which situation they feel similarly, and what helps them in that situation. Together with the coach, they then write down those situations, how they usually behave and what they could try instead. The solutions are found along with the coach. Next time a said situation arises, the child will try to use one of the tricks they have written down. This session aims to teach them emotion regulation and is either applied to the emotion fear or anger. In this way, coaching sessions incorporate everyday challenges brought forward by children and parents to develop context-specific strategies. After four sessions, parents are called to discuss upcoming sessions and to provide guidance on reinforcing EFs at home. The final session serves as a closing session and does not introduce any new topics. Instead, the preceding weeks are reviewed, with each session revisited to discuss the strategies applied as well as any achievements and remaining difficulties. Families are encouraged to continue practising the strategies that have proven effective for them.

##### Analog training

Analogue card games that enhance EF abilities suitable for single or group play. 13 commercially available card games that align with the participants’ ages and require EF skills such as rule retention, flexible adaptation to other players, impulse control and strategic thinking are supplied to the families (see online supplemental table 1). They are able to play at their convenience but are asked to document the frequency and duration of the specific games they played.

### Control group

Participants in the control group receive standard of care. To track and monitor the duration of activities that could enhance EFs, parents and their children are asked to maintain an electronic activity log four times a week for 8 weeks. In the log, participants selected their activities for each 15-minute interval throughout the day from a drop-down menu (sleeping, eating, school lessons, physical education, break time, homework, reading, playing an instrument or singing, watching television, computer games, quiet activities, indoor exercise, outdoor exercise, vigorous indoor exercise, vigorous outdoor exercise, indoor training (in a club), outdoor training (in a club), walking, cycling, public transport, car, other activities). For further specification within these categories, families fill in free text, what those include at the end (see online supplemental table 2). After their participation in the study, families in the control group are offered the gamified computerised training, advice and tips for everyday EF improvement, and the list of analogue games used in E-Fit.

### Feasibility measures

The primary outcome of this feasibility study includes six of eight areas of focus described by Bowen *et al*^51^: acceptability considers suitability, satisfaction and attractiveness; demand assesses how likely the intervention is to be used (actual use and expressed interest); implementation evaluates successful delivery to participants; practicality examines how an intervention can be delivered within available resources and circumstances; integration looks at how well the intervention fits into the family’s home setup; and exploratory efficacy measures its potential success with the intended population. Two other areas of focus*—*adaptation and expansion—were not included, as they pertain to pre-existing, established intervention programmes.

To assess these areas of focus, we used a combination of quantitative and qualitative measures.

Acceptability was evaluated with acceptability targeted questions from the Acceptance and Feasibility Scale (AFS^34^). Although labelled ‘acceptance and feasibility scale,’ these questions covered multiple areas of focus as defined by Bowen *et al*.^51^ To reflect this, we grouped the questions accordingly. It also featured exploratory items allowing participants to indicate which aspects they found most helpful, most enjoyable or least relevant.^34^ Acceptability-targeted questions included questions such as “I liked the online games” or “I felt that the coach contributed to my learning beyond the sessions”. Additionally, participants rated their levels of pleasure and displeasure before and after computerised training sessions using the Children’s Feeling Scale (−5 to 5 scale; online supplemental figure 1).^52^ Coaches rated participants’ observed engagement on a rating sheet during the coaching sessions (low, moderate or high).^53^ Low engagement reflected predominantly passive, distracted and affectively flat behaviour, whereas high engagement reflected focused, effortful, verbally active, persistent and emotionally positive behaviour. Moderate engagement represented mean ratings in the mid-range across these same dimensions, indicating neither predominantly disengaged nor highly engaged behaviour.

Demand was measured with the demand targeted questions from the AFS (eg, “I would do the programme (as a whole) again” or “I would recommend the programme (as a whole) to other children”), as were children’s ratings of how fun and cognitively demanding the computerised training was (Fun and Demand, 1 to 5 scale).^54^ The number of sessions of computerised training, coaching sessions and analogue games completed were also recorded.

Implementation was assessed by monitoring fidelity with the E-Fit Fidelity Measurement System. This assessment was newly developed following existing guidelines.^55^ It evaluated how closely all of the E-Fit components were implemented, including core components, specific questions and session completions, with yes/no response options. For this purpose, the strategy coaching sessions had been recorded.

Practicality was evaluated using practicality targeted questions from the AFS (eg, “Everything worked as it should” or “It was easy to use the online website for the games”), observed coach involvement during coaching sessions (rating sheet, separately rated by two separate raters, 1 to 3 scale,^53^ based on strategy coaching recordings), and the Session Rating Scale after each coaching session (rated by the participants, 0 to 10 scale).^56^ Retention rate served as an additional indicator of practicality, as higher retention suggests that the intervention fits within participants’ available resources and everyday circumstances. Adverse events (AEs) and feedback from coaching sessions were also collected.

Integration was assessed based on integration targeted questions from the AFS such as “A face-to-face meeting with a coach would be better” or “the programme (as a whole) was too long”.

Exploratory efficacy was investigated through baseline, post-intervention and 4-month follow-up assessments of everyday EFs using the BRIEF^42^ and neuropsychological EF performance. All three intervention modalities tapped into the four EF domains including flexibility, working memory, planning and inhibition. To get an exploratory idea about potential efficacy, we have therefore looked at one assessment for each of those domains:

Flexibility: Delis-Kaplan Executive Function System (D-KEFS) Trail Making Test^57^;

Working memory: Wechsler Intelligence Scale for Children-5th edition (WISC-V): Digit Span;^58^

Planning: D-KEFS Tower Task^57^;

Inhibition: D-KEFS Color Word Interference Test^57^; Analyses were conducted with raw scores for all measures.

We also explored the potential effects of the intervention on psychosocial variables gathered at the same timepoints including: The parent-reported Family Relationship Index^59^; self-reported Kidscreen-10 and parent-reported Kidscreen-27^60^; parent-reported Conners-3^61^; parent-reported Social Responsiveness Scale (SRS)^62^; self-reported Resilience Scale (RS-13) completed by both children and parents.^63^ For a detailed rationale behind these measures, see online supplemental 1.

### Descriptive measures

Cardiac and neonatal history was extracted from the participants’ clinical charts. Socioeconomic status (SES) was estimated using a six-point scale for paternal and maternal education, with a cumulative socio-economic status (SES) score ranging from a minimum of 2 to a maximum of 12.^64^ The questionnaire collecting information on parental education was completed by one parent for both parents, regardless of whether there was a single-parent household. Missing values were imputed using multivariate imputation by chained equations. Participants’ IQ was assessed at baseline using a validated short-form of the WISC-V including the subtests Similarities, Matrices, Digit Span and Coding.^39 65^

### Statistical analysis

Descriptive statistics were calculated, including means and SD for continuous variables, medians and IQRs for ordinal variables and frequency counts and percentages for categorical variables.

The Children’s Feeling Scale and the Fun and Demand ratings were collected after each computerised training session, resulting in a densely sampled longitudinal dataset. To assess changes over time while accounting for within-subject correlations, we used linear mixed models (LMMs) with time as a fixed effect and intercept as random effect. This approach allowed us to evaluate overall trends in emotional valence, fun and perceived cognitive demand across the intervention period.

Two raters independently evaluated coaching sessions using the E-Fit Fidelity Measurement System, and inter-rater reliability was reported. Any qualitative data, such as reasons for non-participation and feedback within coaching sessions, were analysed and reported with structured qualitative content analysis.^66^ Initially, main categories were derived inductively from the data. Subsequently, the material was systematically coded. The coding process was iterative, allowing for refinement of the category system. All data were then coded using the final category system, enabling structured comparison and interpretation of relevant content across cases.

To investigate exploratory efficacy on improving EFs and psychosocial variables, analyses of covariance (ANCOVAs) were calculated separately for the post-intervention and the 4-month follow-up outcomes (dependent variables). In each model, group (intervention vs control) was entered as the independent variable, and the corresponding baseline score as a covariate. This approach tests whether the intervention group showed greater improvement than the control group while adjusting for baseline values. The covariate-adjusted means divided by the unadjusted post-intervention/4-month follow-up SD respectively can be interpreted as an adjusted Hedge’s g_A1_: large effect sizes (≥0.80); moderate effect sizes (≥0.50); small effect sizes (≥0.20); negligible effect sizes (<0.20). Accordingly, the reported effect sizes represent the baseline-adjusted between-group difference and can be interpreted as the intervention effect. As no intervention-relevant descriptive variables differed significantly between groups, they were not included as additional covariates in the analysis.

Due to the absence of a total score in the Conners-3 short form, an ordinal linear or quadratic regression was conducted, respectively, using the number of scales that fell outside the normal range as the outcome variable (abnormal scale if T>60; outcome ranges from 0 to ≥4 abnormal scales).

The data were visually inspected in boxplots to identify outliers. Outliers were inspected and kept unless evidence indicated that they were unrelated to the children’s actual cognitive function (eg, hearing aid malfunction during the test, examiner’s mistakes in instructions). Observations with missing values were removed only from analyses involving the respective variable at the respective timepoint, not from the overall dataset; The R statistical software was used for all analyses.

### Patient and public involvement

Children and adolescents with cCHD, their parents and teachers were involved in the intervention development and design. More details about the intervention development can be found in the published protocol (see online supplemental protocol file). Study results will be disseminated to participants and their families through a newsletter and a public information meeting.

## Results

### Population

A total of 60 patients (24% of all eligible patients) agreed to participate and were screened for EF problems and 42 (70% of those screened) were identified with EF problems, as indicated by a BRIEF T-value ≥60 in any of the summary scales, and consequently were enrolled in the study (figure 1). With drop-outs this resulted in a post-intervention sample size of 39 (17 intervention, 22 control group) and a 4-month follow-up sample size of 38 (17 intervention, 21 control group; see also consent rate table 1). Patient characteristics are shown in table 2. The intervention and control groups did not differ in most clinical, demographic or cognitive characteristics. However, children in the intervention group underwent less CPB surgeries and participated more often in regular sports activities than children in the control group.

**Table 1:** Feasibility: areas of focus, assessment tools, results and interpretation

Area of focus	Assessment tool	Group	Timepoint	Completed by	Results Median [IQR] N (%)		Interpretation
**Acceptability**	Acceptance and Feasibility Scale (AFS)^34^ Raw score (1 to 4) ⇑	E-Fit	Last coaching session	Parents Children	3.0 [3.0 to 4.0] 3.0 [3.0 to 4.0] Coaching rated most helpful component: 14 (82%) Coaching rated the most fun component: 8 (47%) Analogue games rated the most fun component: 7 (41%)		**Supportive ✓**
	Children’s Feeling Scale^52^ Raw score (−5 to 5) ⇑	E-Fit	Intervention period, before and after each computerised training	Children	_pre_: 4.0 [3.0 to 5.0] _post_: 4.0 [3.0 to 5.0] slope_post_=0.013, p=0.367		**Supportive ✓**
	Observed engagement^53^ Raw score (1 to 3) ⇑	E-Fit	Each coaching session	Coaches	2.0 [2.0 to 2.3]		Partly supportive
**Demand**	Acceptance and Feasibility Scale^34^ Raw score (1 to 4) ⇑	E-Fit	Final coaching	Parents Children	3.0 [3.0 to 3.0] 3.0 [2.9 to 3.5]		**Supportive ✓**
	Fun and Demand^54^ Raw score (1 to 5) ⇑	E-Fit	Intervention period, before and after each online training	Children	Fun=4.0 [4.0 to 4.0] slope_fun_=0.010, p=0.158 Demand=4.0 [4.0 to 5.0] slope_demand_=−0.002, p=0.779		**Supportive ✓**
	Session data: duration, number of completed sessions	E-Fit	Intervention period				
	Computerised training Completed sessions (1 to 24) ⇑				16.0 [6.0 to 19.0] 16/24 (67%)		Non-supportive
	Coaching Completed coachings (1 to 8) ⇑				8.0 [8.0 to 8.0] 8/8 (100%)		**Supportive ✓**
	Analogue games Raw score ⇑				9.0 [4.0 to 14.0]		Partly supportive
	Consent rate Percentage ⇑	Both	T2		24%		Non-supportive
**Implementation**	E-Fit Fidelity Measurement System tool^55^ Percentage ⇑	E-Fit	T2	Independent raters	Children’s coaching: 100% Parents’ coaching: 90% Inter-rater reliability: perfect agreement		**Supportive ✓**
**Practicality**	Acceptance and Feasibility Scale^34^ Raw score (1 to 4) ⇑	E-Fit	Final coaching	Parents Children	3.0 [2.3 to 3.0] 3.0 [3.0 to 4.0]		**Supportive ✓**
	Feedback in coaching sessions	E-Fit	Each coaching session	Parents Children	See online supplemental 3		Partly supportive
	Observed coach involvement^53^ Raw score (1 to 3) ⇑	E-Fit	T2		3.0 [3.0 to 3.0]		**Supportive ✓**
	Session Rating Scale^56^ Raw score (0 to 10) ⇑	E-Fit	Each coaching session	Children	9.8 [8.6 to 10.0]		**Supportive ✓**
	Retention rate Percentage ⇑	Both	T1, T2		T1: 93% (intervention: 89%; control: 96%) T2: 90% (intervention 89%, control: 91%)		**Supportive ✓**
	Number of adverse events (AEs) Raw score ⇓	Both	Continuously		0		**Supportive ✓**
**Integration**	Acceptance and Feasibility Scale^34^ Raw score (1 to 4) ⇑	E-Fit	Final coaching	Children	3.0 (2.8 to 4.0)		**Supportive ✓**
**Exploratory efficacy**					T1	T2	
	**Flexibility** TMT^57^ Speed ⇓	Both	BL, T1, T2		0.101	0.012	Non-supportive
	**Working memory** Digit span^58^ Number correct (0–54) ⇑	Both	BL, T1, T2		−0.055	−0.050	Non-supportive
	**Planning** Tower Task^57^ Achievement score (0–30) ⇑	Both	BL, T1, T2		0.138	**0.413**	Partly supportive
	**Inhibition** CWIT inhibition^57^ Speed (0–180) ⇓	Both	BL, T1, T2		−0.086	0.063	Non-supportive
	BRIEF parents **BRI**^42^ Raw scores (27–82)⇓	Both	BL, T1, T2		**−0.408**	**−0.903**	**Supportive ✓**
	BRIEF parents **GEC**^42^ Raw scores (72–216) ⇓	Both	BL, T1, T2	Parents*	**−0.237**	**0.631**	**(Partly) supportive ✓**
	BRIEF teachers **GEC**^42^ Raw scores (73–219) ⇓	Both	BL, T1, T2	Teachers	−0.075	0.173	Non-supportive

Scale ranges are omitted if they lack limits. Values are median (IQR). ⇑ indicates higher values representing better outcomes, ⇓ indicates lower values representing better outcomes.

Supportive=supports the intervention group. A result was rated as supportive if it met the following thresholds: for outcomes rated on a 1 to 4 scale, a median rating of ≥3 was considered supportive; for outcomes rated on a 1 to 3 scale, a median rating of 3 was considered supportive; for outcomes rated on a −5 to 5 scale, a median rating of ≥1 was considered supportive; for outcomes rated on a 0 to 10 scale, a median rating of ≥6 was considered supportive. For the consent rate, a threshold of 75% and for the retention rate, a threshold of 90% were defined as supportive. Effect sizes were defined using adjusted Hedge’s gA1: small effects (≥0.2 and <0.5) were rated as partly supportive, and moderate or larger effects (≥0.5) as supportive. All results classified as supportive are shown in bold.

* If possible, completed by both parents separately. Table adapted from Schmid *et al*.^39^

BL, baseline; BRI, Behavioral Regulation Index; BRIEF, Behaviour Rating Inventory of Executive Functions; CWIT, Colour Word Interference Task; EF, executive function; E-Fit, executive function intervention; GEC, global executive composite; T1, post-intervention; T2, 4-month follow-up; TMT, Trail-making Test.

**Table 2:** Patient characteristics

Patient characteristics	Control (n=23)	Intervention (n=19)	P value
Demographics			
Age in years at BL; mean (SD)	11.1 (0.8)	10.9 (0.9)	0.408
Male sex; n (%)	13.0 (56.5)	9.0 (47.4)	0.779
Parental education; median (IQR)	8.0 (8.0−10.0)	8.0 (7.0−10.0)	0.541
Medical history			
Gestational age in weeks; mean (SD)	39.3 (1.6)	38.9 (1.6)	0.438
Prenatal diagnosis; n (%)	6.0 (26.1)	6.0 (31.6)	0.961
Biventricular CHD; n (%)	18.0 (78.3)	17.0 (89.5)	0.579
Univentricular CHD; n (%)	5.0 (21.7)	2.0 (10.5)	0.579
Cyanotic CHD; n (%)	15.0 (65.2)	11.0 (57.9)	0.867
Acyanotic CHD; n (%)	8.0 (34.8)	8.0 (42.1)	0.867
CPB surgeries			
Length of hospital stay after first surgery (days); mean (SD)	29.8 (24.6)	26.4 (22.3)	0.640
Number of total surgeries; median (IQR)	2.0 (1.0–3.0)	1.0 (1.0–2.0)	**0.010**
Cognitive measures at baseline			
IQ at baseline assessment; mean (SD)	97.6 (17.0)	99.3 (13.9)	0.724
BRIEF GEC T-score parent reported values at baseline assessment; mean (SD)	68.2 (8.1)	63.3 (10.6)	0.106
BRIEF GEC T-score teacher reported values at baseline assessment; mean (SD)	57.5 (11.1)	59.5 (11.7)	0.700
Activities at baseline			
Regular sports activities; n (%)	13.0 (56.5)	17.0 (89.5)	**0.044**
Regular music lessons; n (%)	9.0 (39.1)	7.0 (36.8)	1.000
Part of a youth group; n (%)	6.0 (26.1)	4.0 (21.1)	0.986

All results classified as statistically significant (p<0.05) are shown in bold.

BL, baseline; BRIEF, Behavior Rating Inventory of Executive Function; CHD, congenital heart disease; CPB, cardiopulmonary bypass; GEC, global executive composite; IQ, intelligence quotient.

### Results of E-Fit

#### Primary outcome: feasibility

An overview of feasibility results is given in table 1.

Acceptability. Both parents and children rated E-Fit as acceptable on the AFS. The majority of the children indicated that the coaching was the most helpful and most enjoyable component of E-Fit. Analogue games ranked second in terms of most enjoyable. Children’s emotional valence remained high before and after computerised training sessions throughout the whole intervention period. On average, the coaches rated participants’ engagement as moderate.

**Figure 2:**
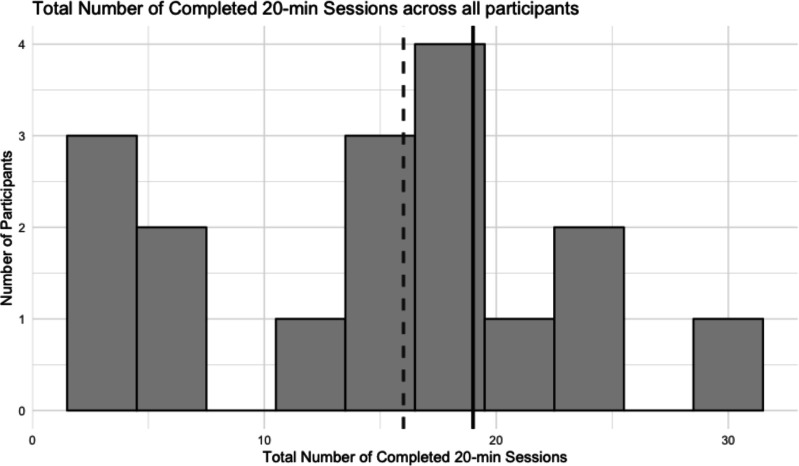
Distribution of the total number of completed 20-minute sessions per participant. Bars represent the number of participants within each session range. The dashed vertical line indicates the median number of completed sessions (median=16.0, IQR=6.0 to 19.0), and the solid vertical line indicates the 80% training completion threshold (~20).

Demand. Parent and child ratings on the AFS indicated a high demand for E-Fit. Children rated the computerised training as fun and cognitively demanding with values ≥4. Four children (24%) completed ≥80% (~20 sessions) of the computerised training as initially targeted (figure 2). All children attended all scheduled child coaching sessions. With the exception of one family, all parents also completed both parent coaching sessions.

Implementation: The coaches closely adhered to coaching guidelines and demonstrated nearly identical levels of involvement. The adherence to the computerised training is described under ‘demand’ above, and problems and successes in implementation are described below in the qualitative analysis.

Practicality of E-Fit on the AFS was high. The detailed qualitative analysis of the participants’ feedback in coaching sessions can be found in online supplemental 2. Participants most frequently reported successful implementation of school-based strategies. Common problems included login difficulties, games not starting and challenges in motivating children to engage with the training. Observed coach involvement during coaching sessions was rated high. In the Session Rating Scale, participants expressed overall satisfaction with the sessions.

The retention rate was high, with the majority of participants returning at T1 and T2.

Integration was supported by high child ratings on the AFS.

**Figure 3:**
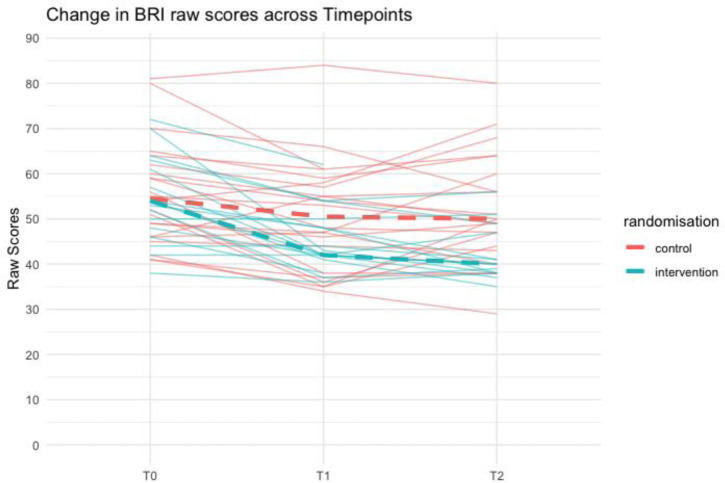
Individual participant trajectories of BRI raw scores across timepoints (**T0, T1, T2**), stratified by randomisation group. Thin lines represent individual participants, and dashed lines indicate group-level median trajectories. BRI, Behavioral Regulation Index.

Exploratory efficacy: Baseline descriptive characteristics of the groups are presented in table 2. For the neurodevelopmental assessment, only negligible effect sizes were observed in the Trail-Making Test as a measure of flexibility, the Digit Span as a measure of working memory and the Colour Word Interference Task as a measure of inhibition. A small effect was observed in the Tower Task at T2. Notably, small and moderate effects were observed in the BRIEF GEC parental report. This was particularly driven by the Behavioral Regulation Index (BRI) of the BRIEF parent-report showing a small effect size for the post-intervention and a large effect size for the 4-month follow-up, both favouring the intervention group (table 1 and figure 3). The detailed results at post-intervention are presented in online supplemental table 3 and at 4-month follow-up in online supplemental table 4.

The detailed results for the secondary outcomes at post-intervention are also presented in online supplemental table 3 and for the 4-month follow-up in online supplemental table 4. Notably, moderate effect sizes were found in the SRS parent-report for the children at post-intervention and at 4-month follow-up, both favouring the intervention group.

#### Activity log control group

Based on the activity logs provided by the control group, we could see that the control group spent a substantial amount of time on calm activities, including board and computer games (see online supplemental figure 2). Looking at the activities presented in online supplemental table 2 more closely, they did not engage in EF-enhancing activities provided to the intervention group.

## Discussion

To the best of our knowledge, this is the first study testing the feasibility of an EF intervention specifically tailored to the needs of patients with cCHD and their families. E-Fit was developed in collaboration with children with cCHD, their parents and teachers, and with healthcare professionals. Our study provides evidence that E-Fit is feasible and suggests EF improvements, particularly in behavioural regulation and effects on social responsiveness. The control group adhered to their treatment, and we can rule out a substantial contamination-induced bias.

In addition, we identified some weaknesses, including limited adherence to the computerised training, moderate engagement in the strategy coaching sessions, and challenges in reaching a high recruitment rate—all of which should be addressed in future iterations of testing the E-Fit intervention.

### Feasibility outcomes

The E-Fit intervention was generally well-received, with participants rating the coaching component as the most helpful and enjoyable aspect. Despite this, engagement during the coaching sessions was only moderate, which indicates a potential for enhancing motivation and active participation. One key factor influencing engagement is autonomy, which plays a crucial role in the development of EFs. Teachers and parents can enhance this process by encouraging independent problem-solving and self-regulation.^53 67^

Children often required parental encouragement to participate in the computerised training, although participants did not report increasing dissatisfaction over time. While previous interventions have employed extrinsic motivators like badges or leaderboards as motivational tools,^68^ families in the needs assessment study expressed concerns about the pressure such performance-driven tools might introduce.^39^ A gamified approach, implementing more appealing in-training incentives, such as building a virtual city^69^ or using avatars and cover stories,^68 70^ could increase children’s commitment to the computerised training.

The CogniFit training component was perceived as both fun and cognitively demanding, which is an ideal combination for EF improvement.^71^ The gamified environment and the adaptive nature of CogniFit promote motivation while ensuring sufficient cognitive challenge, a requirement for durable effects.^40^

However, adherence to the computerised training schedule was inconsistent. A technical issue with the CogniFit software limited session duration to 10 min instead of the intended 20 min, requiring children to complete two consecutive sessions—a requirement not always fulfilled. The limited adherence to this modality may also restrict the interpretability of the feasibility findings related to the computerised training, as these assessments were predominantly completed by participants who engaged in the training.

Implementation fidelity was strong with coaches following the guidelines closely, which increases the reliability and validity of our results.^72^ High fidelity can be ensured in future research by monitoring fidelity regularly and systematically, particularly as multiple coaches will be involved.

In terms of practicality*,* E-Fit was reported as user-friendly and easily integrated into their daily lives and home settings. Participants appreciated the online format of the coaching session. The onset of the COVID-19 pandemic has accelerated the use of telehealth services for patients with cCHD.^73 74^

Participating children’s feedback on the integration of E-Fit into their daily lives was generally positive and they indicated that the skills learnt appeared sustainable.

Exploratory efficacy analyses showed promising results, particularly in parent-reported EFs showing a large effect size for behavioural regulation and a moderate effect size for the general EF scores. This effect was not only detected directly post-intervention, but also at the 4-month follow-up. In the neuropsychologically assessed EFs, only small effects could be found for planning at T2. Thus, the intervention effects were primarily evident in everyday functioning rather than in standardised test performance. The strategy coaching, which showed the highest adherence and therefore the greatest intervention dose, specifically targeted strategies aimed at improving daily functioning. With increased adherence to the other intervention modalities—and consequently a higher overall dose—these effects may eventually also be reflected in standardised test outcomes.

### Strengths and limitations

This study has several strengths. First, the intervention was developed collaboratively with input from patients and their families, teachers and healthcare professionals. The collaboration led to the inclusion of strategy coaching, which was rated the most enjoyable and helpful part of the intervention. Second, the study adhered to a structured framework to collect comprehensive feasibility data and thus facilitate informed decisions about pursuing the intervention in a larger randomised controlled trial to test efficacy. Third, the innovative multimodal approach shows promise in addressing issues of transfer within intervention studies.^75^ However, the study also has several limitations. Although the targeted number of participants was achieved, there was a 76% not-participation rate. Future planning should incorporate systematic communication strategies, using inputs from focus groups.^76^ Partnering with non-governmental organisations (NGOs) and parent associations helps to reach a larger audience, especially families not currently in contact with healthcare providers. SES among participants was relatively high, also compared with the general population in Switzerland.^77^ Previous studies have shown that lower SES is a risk factor for reduced EFs in children with cCHD,^78^ ^79^ even more than perioperative total brain volume or brain injury severity. It is therefore essential to reach families from diverse backgrounds as they may benefit the most from such an intervention.^78^ Families and treating cardiologists could be involved in the development and dissemination of recruitment materials. Technical problems with the CogniFit software allowing only 10 instead of the planned 20 minute sessions lead to incomplete sessions that had to be excluded from adherence analyses, indicating a need for better support and monitoring mechanisms in future iterations. Participants were unblinded after the baseline assessment, potentially introducing biases in subsequent performance and questionnaire responses. Further, some questionnaires were completed in the presence of the investigators/coaches, which may have introduced response bias.

## Conclusions

E-Fit proved feasible and indicated EFs and social responsiveness improvements. The computerised training was perceived as both fun and cognitively demanding, despite variability in adherence. The strategy coaching was rated as particularly fun and helpful. Increasing intrinsic motivation by fostering autonomy in children who showed limited engagement in the coaching sessions may help to improve the efficacy of this intervention. Training coaches, parents and teachers in autonomy-supportive strategies (eg, creating opportunities for choice, pointing out the value of a task) may not only strengthen participant engagement but also boost intrinsic motivation and deepen participants’ understanding of E-Fit’s benefits.^53^

E-Fit was developed specifically for children with cCHD through the active involvement of patients and their parents, allowing us to align the content with their needs and preferences. At the same time, the needs and preferences expressed by patients and parents largely reflected those of children with EF difficulties in general. Therefore, if proven effective in larger and more diverse samples, E-Fit holds promise not only for children with cCHD but also for other clinical populations with similar diffuse EF-related challenges who are largely able to attend regular education, such as children born preterm.^80^

## Supplementary material

10.1136/bmjopen-2025-107681 online supplemental file 1

10.1136/bmjopen-2025-107681 online supplemental file 2

## Data Availability

No data are available.
