# Supplementary material for: Multimodal personalised executive function intervention (E-Fit) for school-aged children with complex congenital heart disease in Switzerland: a randomised controlled feasibility study
Source: BMJ Open. 2026 May 8;16(5):e107681. doi: 10.1136/bmjopen-2025-107681 (PMC13157806; doi:10.1136/bmjopen-2025-107681)
Supplement: online supplemental file 2 [file bmjopen-16-5-s002.pdf]

Supplementary 1: Rationale Secondary Outcomes

The Family Relationship Index (FRI)

The FRI has emerged as a significant tool for assessing family dynamics and their impact on various psychological outcomes, including EFs. The FRI evaluates key dimensions of family relationships, such as cohesion, expressiveness, and conflict, which are critical for understanding how family environments influence individual development and functioning [1]. A previous study has shown that positive family functioning correlates with higher levels of EFs in children, suggesting that supportive family environments foster cognitive skills essential for self-regulation and problem-solving [2], which is why we decided to include the FRI as a secondary outcome measure.

Kidscreen-10 and Kidscreen-27

An association between EFs and quality of life has been found in healthy children and adults [3–5]. Also in other populations such as participants with epilepsy, brain tumours or attention deficit hyperactivity disorder (ADHD; [6–8]), and in children with CHD, this connection has been found [9–11]. The Kidscreen-10 and Kidscreen-27 were developed for the Kidscreen project to standardize quality of life assessment for children and adolescents in national and European health surveys. It also serves as a tool for evaluating the quality of life of chronically ill youth [12]. Therefore we included this as a secondary outcome measure, to see potential effects of E-Fit onto health-related quality of life.

Conners-3

The Conners-3 is a clinical questionnaire method for the assessment of ADHD [13]. Previous studies indicate that executive dysfunction is prevalent among children with ADHD, as evidenced by studies that highlight significant correlations between ADHD symptoms and deficits in EFs, such as poor organization and difficulties in initiating tasks [14,15]. Further, in a previous study with children with CHD using Cogmed© working memory training, improvements in parental ratings of inattention have been observed [16]. The Conners-3 is a widely used tool for evaluating ADHD symptoms and research shows that targeted interventions aimed at improving EFs can lead to meaningful changes. Therefore, we decided to include the Conners-3 as a secondary outcome measure in our study.

Social Responsiveness Scale (SRS)

The SRS is widely recognized as a valid measure for assessing social, communicative and rigid behaviour in children and adolescents in the sense of a dimensional diagnosis of autism. There is a

high degree of convergence with autism spectrum disorders in the extreme range of the scale [17]. Research indicates that behavioral regulation processes, such as inhibition, shifting, and emotional control, are predictive of social functioning across both children with autism spectrum disorder and typically developing peers [18]. This suggests that deficits in EFs may contribute to social impairments, as individuals with lower EF skills often exhibit heightened difficulties in social interactions [18]. Additionally, interventions aimed at enhancing EFs have shown promise in improving social skills, suggesting a bidirectional relationship where better EFs can lead to improved social responsiveness [19,20]. Therefore, we decided to include the SRS as a secondary outcome measure.

Resilience Scale (RS-13)

The RS-13 measures the construct resilience in the sense of psychological persistence [21]. Resilience is defined as the ability to adapt positively in the face of adversity, while EFs encompass cognitive processes such as inhibition, working memory, and cognitive flexibility, which are crucial for goal-directed behaviour and emotional regulation. A growing body of literature suggests that these constructs are interrelated [22,23]. Therefore, enhancing EFs can lead to improved resilience, suggesting that interventions aimed at improving EFs may also foster resilience in vulnerable populations, which was the reason for us to include the RS-13 as a secondary outcome measure.

Supplementary 2: Qualitative analysis

The following descriptive synthesis presents feedback from the coaching sessions summarized into categories; it is not intended to be quantified and should not be interpreted as such. The qualitative analyses were conducted by the first author (AS), following established procedures for structured qualitative content analysis.

General Feedback

Parents have reported that implementing newly learned strategies requires training, which can be difficult due to busy schedules. Maintaining consistency can also be challenging for the child.

Demand

Parents were grateful for the opportunity provided, but they reported that their child was facing challenges in implementing some of the strategies suggested. Although the child enjoyed the online games included in the experience, they were uncertain about its overall helpfulness despite appreciating the different aspects covered.

Adherence

The main reason behind not implementing the strategies that were discussed was the fact that the situations which were prepared and discussed earlier did not occur frequently, the card games were not played often and the stickers were hardly ever used.

Successes

The strategies intended for school use were most frequently implemented, facilitating the child's self-reflection and highlighting where parents could support.

Problems

The most common issues reported by users were difficulties with logging in or the games failing to start after logging in. Furthermore, motivating children to participate in the computerized training was often a challenge, requiring some persuasion. The online training was occasionally forgotten during holidays. Additionally, some of the games, such as Cube Foundry and Bee Balloon, were found to be too challenging for some children, potentially leading to frustration.

Supplementary Table 1.

Game	Trained skills			
	flexibility	working memory	planning	inhibition
Gold (Game Factory ®)	x	x	x	
Dodelido ("Drei Magier ®")	x	x		x
Särge schubsen ("Drei Magier ®")	x	x	x	x
Geistesblitz 2.0 ("Zoch")	x	x		x
Beaver Gang ("Amigo Spiele")	x	x	x	
Beaver Clan ("Amigo Spiele")	x	x	x	
Memo Dice ("Amigo Spiele")	x	x	x	
5er finden (HABA ®)	x		x	
The nasty 7 ("Drei Hasen in der Abendsonne")	x	x		x
Yokai (Game Factory ®)	x	x	x	
Okiya ("Pegasus Spiele")	x		x	
Perplexus Crazy run (2 PE Original, 5 FT 208, 6 PE Epic)				x
Thunder Pops				x

Games selected based on Spielliste Titelblatt 1.5. (spielendfoerdern.ch)

Supplementary Table 2. Detailed activities of the control group.

Reading, audiobooks	Television	Gaming	Internet	Spiele
Magazines, The three ???, Non-fiction books, School of magical animals, Carlotta, Diary of a wimpy kid, Mangas, Harry Potter, Lucky Luke, Lustiges Taschenbuch, Woodwalkers, Lotta Leben, Percy Jackson, The Little Prince, Wozzle Gozzle, Petronella Apfelmus, Globi, die 3 Rabauken	Lotta Leben, Bibi und Tina, Mädchen-WG, Berlin School of Arts, Logo, Sport, Das grosse Backen, Top Gun, Star Wars, Harry Potter, Disney Movies, Documentaries, Home alone 2	Lenas Ranch, Burger belegen, FIFA, Minecraft, Need for speed, Lego Star Wars, Fortnite, Animal crossing, Brawlstar, Clash of Clans, Mario Kart	meinklett Portal, kicker.de, Youtube	Playmobil, Tipp-Kick, Schach, Basteln, zeichnen, ausmalen, Monopoly, Spiel des Lebens, UNO, Fisher Technik, Gravi Track, Tiptoi Wetter, Eile mit Weile

Supplementary Table 3. Impact of E-Fit on improving EF skills T1 raw scores.

Variable, Range	E-Fit		Control group		Effect sizes
**Neuropsychological assessment**	**BL (n = 17)**	**T1 (n = 17)**	**BL (n = 22)**	**T1 (n = 22)**	
**CWIT inhibition** [24]<br>Speed (0-180) ⇓	84.0 [72.0 to 91.0]	69.0 [59.0 to 77.0]	77.0 [70.0 to 95.5]	75.0 [64.3 to 83.5]	-0.086
**TMT** [24]<br>Speed ⇓	93.0 [79.0 to 126.0]	78.0 [68.0 to 130.0]	87.5 [68.5 to 129.5]	78.0 [68.5 to 129.5]	0.101
**Tower Task** [24]<br>Achievement Score (0-30) ⇑	15.6±2.9	18.2±3.2	14.8±3.5	17.7±3.5	0.138
**Digit span** [26]<br>Number Correct (0-54) ⇑	22.0 [20.0 to 26.0]	21.0 [19.0 to 26.0]	21.0 [16.0 to 24.0]	22.0 [18.0 to 27.0]	-0.055
**Questionnaires**					
**BRIEF parents BRI** [28]<br>Raw scores (27-82) ⇓	54.0 [48.0 to 61.0]	42.0 [42.0 to 48.0]	54.5 [46.8 to 61.5]	50.5 [39.5 to 57.8]	**-0.408**
**BRIEF parents MI** [28]<br>Raw scores (44-132) ⇓	90.7±18.1	82.3±19.5	98.7±17.4	93.0±22.2	-0.145
**BRIEF parents GEC** [28]<br>Raw scores (72-216) ⇓	144.9±24.2	127.3±24.5	154.5±23.3	143.5±32.1	**-0.237**
**FRI** [1]<br>Raw score (0-27) ⇑	12.0 [10.0 to 13.0]	12.0 [9.0 to 15.0]	11.0 [6.0 to 12.8]	12.0 [9.3 to 14.0]	-0.029
**Kidscreen-10** [12]<br>T-scores (0-70) ⇑	50.0 [36.3 to 57.8]	47.7 [43.2 to 55.4]	51.4 [41.0 to 55.4]	50.6 [41.0 to 56.6]	-0.101
**Kidscreen-27** [12]<br>T-scores (0-80) ⇑	47.5 [42.8 to 49.3]	48.0 [44.8 to 53.0]	45.0 [40.0 to 50.0]	44.0 [41.5 to 46.0]	**0.415**
**Conners-3 Number of abnormal subscales** [13]<br>Raw scores (0-6) ⇓	4.0 {1.0}	2.0 {1.0}	4.0 {1.8}	4.0 {2.0}	**0.32 (0.07 – 1.54)***
**SRS** [17]<br>T-scores (16-100) ⇑	59.0 [48.5 to 66.0]	55.0 [47.0 to 62.5]	67.0 [58.3 to 70.8]	63.5 [56.3 to 66.5]	**-0.427**
**RS-13 children** [21]<br>Raw score (13-91) ⇑	69.0 [66.0 to 74.0]	65.0 [61.0 to 79.0]	68.0 [58.0 to 76.0]	69.0 [65.0 to 77.5]	-0.095
**RS-13 parents** [21]<br>Raw score (13-91) ⇑	69.8±11.4	76.2±9.5	76.4±7.5	73.3±9.2	**0.701**

*Note: BL = Baseline; T1 = post-intervention; CWIT = Colour Word Interference Task, TMT = Trail-making Test, BRIEF = Behaviour Rating Inventory of Executive Functions, Behavioral Regulation Index, MI = Metacognition Index, GEC = Global Executive Composite, FRI = Family Relationship Index; SRS = Social Responsiveness Scale; RS-13 = Resilience Scale-13. Scale ranges are omitted if they lack limits. Values are n (%), mean±SD, median [interquartile range], or modus {IQR} depending on whether the data is normally distributed or not. Adjusted Hedge's g for the ANCOVA were calculated according to Hedges et al. [29], is reported as an effect size and can be interpreted as Hedge's g: moderate to high effect sizes (≥ 0.50); small effect sizes (≥ 0.20); negligible effect sizes (< 0.20). *OR (95% CI). All interpretable effect sizes are bold. ⇑ indicates higher values representing better performance, ⇓ indicates lower values representing lower values.*

Supplementary Table 4. Impact of E-Fit on improving EF skills T2 raw scores.

Variable, Range	E-Fit		Control group		Effect sizes
**Neuropsychological assessment**	**BL (n = 17)**	**T2 (n = 17)**	**BL (n = 22)**	**T2 (n = 21)**	
**CWIT inhibition** [24]<br>Speed (0-180) ⇓	84.0 [72.0 to 91.0]	66.0 [58.0 to 73.0]	77.0 [70.0 to 95.5]	67.0 [60.0 to 78]	0.063
**TMT** [24]<br>Speed ⇓	93.0 [79.0 to 126.0]	86.0 [63.0 to 97.0]	87.5 [68.5 to 129.5]	60.0 [53.0 to 73.0]	0.012
**Tower Task** [24]<br>Achievement Score (0-30) ⇑	15.6±2.9	20.0±2.9	14.8±3.5	18.3±3.8	**0.413**
**Digit span** [26]<br>Number Correct (0-54) ⇑	22.0 [20.0 to 26.0]	21.0 [19.0 to 26.0]	21.0 [16.0 to 24.0]	22.0 [18.0 to 27.0]	-0.050
**Questionnaires**					
**BRIEF parents BRI** [28]<br>Raw scores (27-82) ⇓	54.0 [48.0 to 61.0]	40.0 [38.0 to 44.0]	54.5 [46.8 to 61.5]	50.0 [44.0 to 60.0]	**-0.903**
**BRIEF parents MI** [28]<br>Raw scores (44-132) ⇓	90.7±18.2	78.9±18.4	98.7±17.4	95.1±19.9	**-0.422**
**BRIEF parents GEC** [28]<br>Raw scores (72-216) ⇓	144.9±24.2	120.9±23.4	154.5±23.3	147.2±29.6	**-0.631**
**FRI** [1]<br>Raw score (0-27) ⇑	12.0 [10.0 to 13.0]	13.5 [10.5 to 15.5]	11.0 [6.0 to 12.8]	13.0 [11.0 to 14.0]	-0.135
**Kidscreen-10** [12]<br>T-scores (0-70) ⇑	50.0 [36.3 to 57.8]	51.8 [43.8 to 57.1]	51.4 [41.0 to 55.4]	52.2 [42.4 to 59.0]	0.019
**Kidscreen-27** [12]<br>T-scores (0-80) ⇑	47.5 [42.8 to 49.3]	50.0 [43.8 to 52.8]	45.0 [40.0 to 50.0]	47.0 [41.0 to 49.0]	**0.573**
**Conners-3 Number of abnormal subscales** [13]<br>Raw scores (0-6) ⇓	4.0 {1.0}	1.0 {3.0}	4.0 {1.8}	4.0 {2.0}	**0.24 (0.04 – 1.36)***
**SRS** [17]<br>T-scores (16-100) ⇑	59.0 [48.5 to 66.0]	55.0 [48.0 to 65.0]	67.0 [58.3 to 70.8]	63.0 [56.0 to 69.3]	**-0.521**
**RS-13 children** [21]<br>Raw score (13-91) ⇑	69.0 [66.0 to 74.0]	71.5 [63.3 to 79.0]	68.0 [58.0 to 76.0]	71.5 [67.3 to 77.3]	0.015
**RS-13 parents** [21]<br>Raw score (13-91) ⇑	69.8±11.4	72.0±7.6	76.4±7.5	76.3±8.1	0.093

*Note: BL = Baseline; T2 = 4-month follow-up; CWIT = Colour Word Interference Task, TMT = Trail-making Test, BRIEF = Behaviour Rating Inventory of Executive Functions, Behavioral Regulation Index, MI = Metacognition Index, GEC = Global Executive Composite, FRI = Family Relationship Index; SRS = Social Responsiveness Scale; RS-13 = Resilience Scale-13. Scale ranges are omitted if they lack limits. Values are n (%), mean±SD, median [interquartile range], or modus {IQR} depending on whether the data is normally distributed or not. Adjusted Hedge's g for the ANCOVA were calculated according to Hedges et al. [29], is reported as an effect size and can be interpreted as Hedge's g: moderate to high effect sizes (≥ 0.50); small effect sizes (≥ 0.20); negligible effect sizes (< 0.20). *OR (95% CI). All interpretable effect sizes are bold. ⇑ indicates higher values representing better performance, ⇓ indicates lower values representing lower values.*

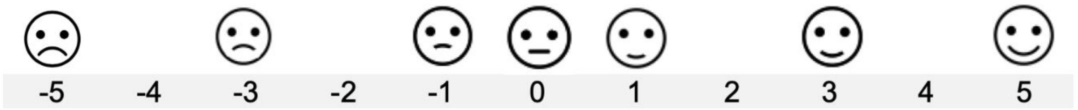

Supplementary Figure 1. Children's Feeling Scale. A numerical scale for children to state their current emotional valence, indicating levels of pleasure and displeasure. Children are instructed to "Circle a number" corresponding to how they feel at the moment.

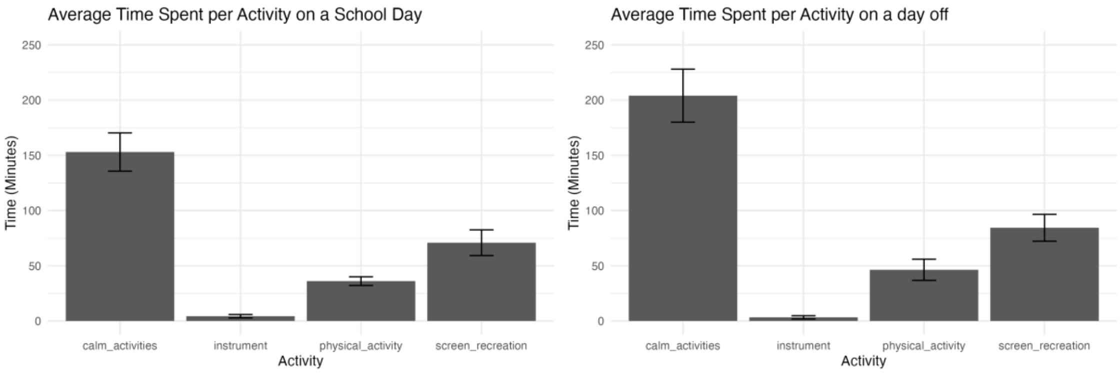

Supplementary Figure 2. Note: calm activities = reading, other quiet activities or games such as listening to music, audiobooks, drawing, painting, handicrafts, or board games. Instrument: playing an instrument or singing. Physical activity = Physical education, running, ball games (not in a sports club) indoors or outdoors, training, horse riding, or ballet (in a sports club) indoors or outdoors; screen recreation = television and computer games.

References:

1 Hoge RD, Andrews DA, Faulkner P, et al. The family relationship index: Validity data. Journal of Clinical Psychology. 1989;45:897–903. doi: 10.1002/1097-4679(198911)45:6<897::AID-JCLP2270450611>3.0.CO;2-T

2 Blair C, Raver CC, Berry DJ. Two Approaches to Estimating the Effect of Parenting on the Development of Executive Function in Early Childhood. Dev Psychol. 2014;50:554–65. doi: 10.1037/a0033647

3 Davis JC, Marra CA, Najafzadeh M, et al. The independent contribution of executive functions to health related quality of life in older women. BMC Geriatr. 2010;10:16. doi: 10.1186/1471-2318-10-16

4 Huang C-C, Lu S, Rios J, et al. Associations between Mindfulness, Executive Function, Social-Emotional Skills, and Quality of Life among Hispanic Children. International Journal of Environmental Research and Public Health. 2020;17:7796. doi: 10.3390/ijerph17217796

5 Laera G, Joly-Burra E, Zuber S, et al. Do executive functions explain older adults' health-related quality of life beyond event-based prospective memory? Aging, Neuropsychology, and Cognition. 2023;30:135–49. doi: 10.1080/13825585.2021.1989368

6 Cantisano N, Menei P, Roualdes V, et al. Relationships between executive functioning and health-related quality of life in adult survivors of brain tumor and matched healthy controls. Journal of Clinical and Experimental Neuropsychology. 2021;43:980–90. doi: 10.1080/13803395.2022.2040432

7 Matza LS, Rentz AM, Secnik K, et al. The link between health-related quality of life and clinical symptoms among children with attention-deficit hyperactivity disorder. Journal of Developmental & Behavioral Pediatrics. 2004;25:166–74.

8 Sherman EMS, Slick DJ, Eyrl KL. Executive Dysfunction Is a Significant Predictor of Poor Quality of Life in Children with Epilepsy. Epilepsia. 2006;47:1936–42. doi: 10.1111/j.1528-1167.2006.00816.x

9 Marino BS, Cassedy A, Drotar D, et al. The Impact of Neurodevelopmental and Psychosocial Outcomes on Health-Related Quality of Life in Survivors of Congenital Heart Disease. The Journal of Pediatrics. 2016;174:11-22.e2. doi: 10.1016/j.jpeds.2016.03.071

10 Neal AE, Stopp C, Wypij D, et al. Predictors of Health-Related Quality of Life in Adolescents with Tetralogy of Fallot. The Journal of Pediatrics. 2015;166:132–8. doi: 10.1016/j.jpeds.2014.09.034

11 Sanz JH, Wang J, Berl MM, et al. Executive Function and Psychosocial Quality of Life in School Age Children with Congenital Heart Disease. The Journal of Pediatrics. 2018;202:63–9. doi: 10.1016/j.jpeds.2018.07.018

12 Ravens-Sieberer U. The KIDSCREEN questionnaires. Quality of life quiestionners for children and adolescents - handbook. 2006.

13 Lidzba K, Chrstiansen H, Drechsler R. Conners Skalen zu Aufmerksamkeit und Verhalten - 3. Deutschsprachige Adaptation der Conners 3rd Edition® (Conners 3®) von C. Keith Conners. 2013.

14 Miklós M, Futó J, Komáromy D, et al. Executive Function and Attention Performance in Children with ADHD: Effects of Medication and Comparison with Typically Developing Children. Int J Environ Res Public Health. 2019;16:3822. doi: 10.3390/ijerph16203822

15 Silverstein MJ, Faraone SV, Leon TL, et al. The Relationship Between Executive Function Deficits and DSM-5-Defined ADHD Symptoms. J Atten Disord. 2020;24:41–51. doi: 10.1177/1087054718804347

16 Calderon J, Bellinger DC, Hartigan C, et al. Improving neurodevelopmental outcomes in children with congenital heart disease: protocol for a randomised controlled trial of working memory training. BMJ Open. 2019;9:e023304. doi: 10.1136/bmjopen-2018-023304

17 Bölte S, Poustka F. Skala zur Erfassung sozialer Reaktivität - Dimensionale Autismus-Diagnostik. Deutsche Fassung der Scoial Responsiveness Scale (SRS) von John N. Constantino und Christian P. Gruber. 2007.

18 Leung RC, Vogan ,Vanessa M., Powell ,Tamara L., et al. The role of executive functions in social impairment in Autism Spectrum Disorder. Child Neuropsychology. 2016;22:336–44. doi: 10.1080/09297049.2015.1005066

19 Gardiner E, Iarocci G. Everyday executive function predicts adaptive and internalizing behavior among children with and without autism spectrum disorder. Autism Research. 2018;11:284–95. doi: 10.1002/aur.1877

20 Kenworthy L, Anthony LG, Naiman DQ, et al. Randomized controlled effectiveness trial of executive function intervention for children on the autism spectrum. Journal of Child Psychology and Psychiatry. 2014;55:374–83. doi: 10.1111/jcpp.12161
21 Leppert K, Koch B, Brähler E, et al. Resilience scale - evaluation of a long (RS-25) and a short version (RS-13). Klinische Diagnostik und Evaluation. 2008.
22 Zhang Y, Zhang X, Zhang L, et al. Executive Function and Resilience as Mediators of Adolescents' Perceived Stressful Life Events and School Adjustment. Front Psychol. 2019;10. doi: 10.3389/fpsyg.2019.00446
23 Zhou Y, Yu NX, Liang Z, et al. Preadolescents' executive functions and resilience development: A cascade model of resilience resources and resilient functioning. Journal of Adolescence. 2024;96:1929–41. doi: 10.1002/jad.12392
24 Delis DC, Kaplan E, Kramer JH. Delis-Kaplan Executive Function System. APA PsycNet. 2001. https://psycnet.apa.org/doiLanding?doi=10.1037%2Ft15082-000 (accessed 19 January 2022)
25 Zimmermann P, Fimm B. Testbatterie zur Erfassung von Aufmerksamkeitsstörungen (TAP 2.3.). 2012.
26 Wechsler D. Study Citation Wechsler, D. (2014). WISC-V: Technical and Interpretive Manual. Bloomington. 2014.
27 Lejuez CW, Aklin W, Daughters S, et al. Reliability and Validity of the Youth Version of the Balloon Analogue Risk Task (BART–Y) in the Assessment of Risk-Taking Behavior Among Inner-City Adolescents. Journal of Clinical Child & Adolescent Psychology. 2007;36:106–11. doi: 10.1080/15374410709336573
28 Drechsler R, Steinhausen H-C. Verhaltensinventar zur Beurteilung exekutiver Funktionen BRIEF. Deutschsprachige Adaption des Behavior Rating Inventory of Executive Function. Published Online First: 2013.
29 Fritz C, Morris P, Richler J. Effect Size Estimates: Current Use, Calculations, and Interpretation. Journal of experimental psychology General. 2011;141:2–18. doi: 10.1037/a0024338
