# Supplementary material for: Multimodal personalised executive function intervention (E-Fit) for school-aged children with complex congenital heart disease in Switzerland: a randomised controlled feasibility study
Source: BMJ Open. 2026 May 8;16(5):e107681. doi: 10.1136/bmjopen-2025-107681 (PMC13157806; doi:10.1136/bmjopen-2025-107681)
Supplement: Supplementary file 3 [file bmjopen-2025-107681.reviewer_comments.pdf]

PEER REVIEW HISTORY

BMJ Open publishes all reviews undertaken for accepted manuscripts. Reviewers are asked to complete a checklist review form and are provided with free text boxes to elaborate on their assessment. These free text comments are reproduced below.

ARTICLE DETAILS

Title (Provisional)

Original research: Multimodal personalised executive function intervention (E-Fit) for school-aged children with complex congenital heart disease in Switzerland: A randomised controlled feasibility study

Authors

Schmid, Alenka Sarah; Ehrler, Melanie; Bunge, Silvia; Kretschmar, Oliver; Landolt, Markus; Rousson, Valentin; Tuura, Ruth O'Gorman; Wehrle, Flavia; Latal, Beatrice

---

VERSION 1 - REVIEW

**Reviewer**	**1**
**Name**	**Williams, Tricia**
**Affiliation**	**The Hospital for Sick Children**
**Date**	**04-Sep-2025**
**COI**	**None**

Thank you for inviting me to review this manuscript. I commend the manuscript's innovative approach and recognition of the value of its contributions to neuropsychological interventions for youth with cCHD, particularly the multimodal and family informed approach. However, revisions are required to enhance clarity, methodological rigor, and interpretability. Key concerns include insufficient detail regarding feasibility and participant characteristics, an overabundance of exploratory outcomes relative to sample size, lack of clarity in statistical and qualitative analyses, and insufficient synthesis of results and discussion. Addressing these issues by focusing on core outcome variables, improving the presentation and justification of tables and figures, and clarifying intervention tailoring will be helpful to strengthening the manuscript and supporting its contribution to the field.

I provide detailed recommendations for the author's consideration by section below.

Abstract:

• Clearly describe feasibility measures.

• Briefly report participant characteristics, including mean age, age range, and sex.

• Specify whether analog games were played a median of nine times per week or in total.

• Explain how practicality is measured by retention.

• Clarify the connection between social responsiveness and the intervention as an exploratory efficacy outcome.

• Outline the multimodal nature of the intervention and identify which modalities are used.

Introduction

The introduction paragraph provides a clear rationale for the study. In the review of prior studies, it would be helpful to specify how efficacy was measured—whether by parent report, clinician ratings, performance-based scores, or other methods—as the list of studies suggests a mix of approaches. Clarification in this section would improve transparency.

At the end of page 5, the authors summarize literature supporting multimodal interventions. An additional sentence might explicitly clarify whether these interventions involve combining tasks targeting multiple executive function domains.

On page six, line 5, the manuscript notes that the present study includes a comprehensive needs assessment. It may enhance clarity to rephrase this to indicate that the current study was informed by such an assessment, as the details are not presented within the manuscript aside from the review.

It would be useful to understand the authors' rationale for assessing feasibility alongside conducting a randomized controlled trial (RCT). Was the feasibility of E fit previously piloted before the RCT? If not, testing both the intervention's feasibility and its implementation within an RCT may introduce potential confounds.

Methods & Analyses

Please clarify the rationale for stratifying by maternal education. Did the authors consider randomizing participants according to the severity of executive functioning concerns?

How did participants respond to questions assessing feasibility? Was this interaction conducted with therapists, and if so, was it online? Is there any potential bias among therapists, particularly regarding the therapeutic relationship?

The authors are commended for their efforts in assessing possible contamination and acknowledging other activities that foster executive functioning. Could specific examples be provided of the computerized training methods and particular components of executive functioning targeted both within the program and during coaching sessions?

The authors refer to a manual standardizing the coaching sessions, which appears to be Dawson and Guare's executive functioning workbook (Smart but Scattered). How was this resource leveraged and adapted for the study?

Were coaching sessions recorded for coding or fidelity assessment purposes?

While it is acknowledged that efficacy measures are exploratory, I recommend narrowing the range of outcomes analyzed and directly linking them to executive functioning domains

addressed by the interventions. If the intervention is personalized, changes would only be expected in targeted domains, making analysis complex. Consider focusing on core or primary outcomes, as the current sample size may not support the number of variables included.

Under descriptive measures, please clarify the protocol followed in cases where only one source of education was reported, or in single-parent households.

A notable strength of the analysis is the capacity to observe trends and within-subject ratings across the intervention period.

Please specify how coaching sessions were rated—were assessments based on video recordings or did multiple evaluators participate in sessions?

The statistical approach is appropriate; however, the study appears underpowered for the number of comparisons made. As noted above, I recommend prioritizing and possibly grouping outcome types to ensure analyses are clearly tied to intervention goals and rationales. Currently, outcome measures seem broadly associated with executive functioning without a consistent connection to the intervention itself.

Results

The consent rate is 24%.

There are several "error reference not found" notices in the results section, beginning at the top of page 11. There is also inconsistent capitalization, for example, in the tables.

The authors mention qualitative analysis of participant feedback, but the specific qualitative analytical approach is not described in the analysis section.

Retention was high in some aspects of the program; however, only four children completed 80% of the computerized training, which may indicate limited fidelity or acceptability for this modality.

Regarding the number of analyses conducted for the exploratory results, the presentation in the results section could be improved. As previously suggested, focusing on two to three key outcome variables and presenting their effect sizes in the results would strengthen the section. The manuscript refers too frequently to supplementary tables; it is recommended to identify core tables for inclusion in the main results section and to report relevant statistics.

Discussion

The authors introduce a creative and original intervention; however, it remains unclear at this stage how the approach was specifically tailored to the needs of patients with CHD, how it might differ for individuals with other medical conditions, or whether the customization is solely based on the unique executive functioning challenges of each child.

It is challenging to reconcile reports describing the cognitive training component as both enjoyable and demanding with the observed low rate of completion among children in this

segment. Reference is made to the small number of participants who completed at least 80% of these sessions.

The discussion could be strengthened by providing a more coherent and concise summary of the results section, particularly after further revisions to that section are made. Currently, some results referenced in the discussion are only found within supplementary analyses sections.

While the study offers a comprehensive overview of its limitations, the specific challenges identified among children and families from lower SES backgrounds remain unclear, as these were not reflected in the results. Generally, there appeared to be difficulties engaging children in certain aspects of the intervention, but a direct connection to SES was not evident. If SES is indeed a factor, this should be substantiated within the results section. The only significant differences highlighted pertained to surgeries involving cardiopulmonary bypass and participation in sports activities.

The authors are advised to moderate their conclusions regarding the efficacy of the intervention. It may be beneficial to establish a priori feasibility metrics to confidently assert the feasibility of the intervention period; at present, concerns regarding adherence and participation persist and warrant cautious interpretation.

Table 1

The brief GEC parent report difference between groups is concerning; future randomization should address this through stratified methods.

Table 2 is useful, but the results should be more synthesized and clearer when presenting percentiles, means, or medians. A formal definition of "supportive" and "non-supportive" would also strengthen the authors' interpretation.

Separate the primary feasibility outcomes into a single table. The current presentation of exploratory efficacy variables is too vague; it's not appropriate to group entire measures like WISC or DKEFS together. Specify the number of analyses, variables, and relevant indices for each subtest.

Figure 1

Can the author explain why so many were unreachable? Is this common in their clinical follow-up for congenital heart disease?

What is the purpose of Figure 4? It includes data for four participants?

Page 27 lacks clarity regarding whether it references an appendix or a justification of measures. There should be a clear presentation of stratified outcomes and screening tools.

The qualitative analysis appears descriptive; was the interview data synthesized in terms of percentages of families reporting opportunities across indices? Please clarify.

It's unclear how "calm activities" differ from EF-enhancing activities if they aid behaviour regulation.

Supplemental Table 3 lists about 16 neuropsychological test outcomes but omits supplementary questionnaire variables shown in Supplemental Table 1.

**Reviewer**	**2**
**Name**	**Gallagher, Anne**
**Affiliation**	**Université de Montréal**
**Date**	**17-Nov-2025**
**COI**	**None**

This is a very well-written manuscript and an extremely important and relevant contribution given the frequent executive function challenges in children with CHD and their impact on daily life and quality of life. My comments and suggestions are generally minor but hopefully will help to clarify certain points.

Abstract

In the Interventions section, it is not clear whether there are 2, 3, or 4 groups. This point should be clarified. In Section 2.2.1, it is very clear that the study included 2 groups.

Methods and Results

Why was the randomization stratified by maternal education? This is interesting.

How was the 4-month duration for the long-term follow-up determined? I recommend justifying this in the manuscript. Is there evidence that 4 months post-intervention is sufficient to demonstrate a long-term or lasting effect of the intervention?

How were the activities presented in Supplementary Table 2 selected?

It is mentioned for several measures that they were evaluated “based on integration targeted questions from the AFS”. For readers who are not familiar with this scale (myself included), it would be helpful to provide one or more examples of the AFS questions used each time this is mentioned.

“Analyses were conducted with raw scores for all measures.” Why were raw scores used instead of standard scores? How is the effect of age accounted for when using raw scores? Was age controlled for in the analyses?

I am not sure about all the measures, but repeating certain WISC-V subtests after 4 months will almost certainly introduce a practice effect. What is the potential impact of this on the results?

Discussion

In the first paragraph of the Discussion, the authors state: "...suggest EF improvements particularly in behavioral regulation and effect on social responsiveness." It is not clear to me which specific results support this claim.

Few technical issues are reported in the manuscript. Will these technical issues be addressed in the next version of the intervention?

The exploratory analyses for EF improvements between BL and T1 and T2 (Suppl. Tables 3 and 4) are interesting. Although improvements appear greater in the Intervention group, some improvements are also present in the Control group for certain measures. Are the effect sizes reported only for the Intervention group? Did the authors compare the magnitude of improvement in the intervention group versus the control group?

What are the next steps? Another feasibility study to improve recruitment rate, address technical issues, etc.? Or a transition to a full RCT? Did this feasibility study aim to establish the sample size for a future full RCT?

Minor comments :

- Pages 9, 10 and 11 "Error! Reference source not found" – please add the references (3)

- Page 12: "The CogniFit training component was perceived as both fun and cognitively demanding, which is an ideal combination for EF improvement. " Please provide a reference.

- Supplementary Table.2 There are some typos and German words.

- Page 13, line 2: replace . by ,

VERSION 1 - AUTHOR RESPONSE

<u>Reviewer #1:</u>
Dr. Tricia Williams, The Hospital for Sick Children
Comments to the Author:

Thank you for inviting me to review this manuscript. I commend the manuscript's innovative approach and recognition of the value of its contributions to neuropsychological interventions for youth with cCHD, particularly the multimodal and family informed approach. However, revisions are required to enhance clarity, methodological rigor, and interpretability. Key concerns include insufficient detail regarding feasibility and participant characteristics, an overabundance of exploratory outcomes relative to sample size, lack of clarity in statistical and qualitative analyses, and insufficient synthesis of results and discussion. Addressing these issues by focusing on core outcome variables, improving the presentation and justification of tables and figures, and clarifying intervention tailoring will be helpful to strengthening the manuscript and supporting its contribution to the field.
I provide detailed recommendations for the author's consideration by section below.
Response: We thank you for your appreciation of the intervention and for the critical comments that we have addressed point-by-point below.

1. Abstract:
• Clearly describe feasibility measures.

Response: Thank you for this valuable comment. We have revised the abstract so that the feasibility measures reported in the results section are now described in more detail in the methods section. Despite focusing only on those reported in the abstract, the abstract now encompassing 369 words. We leave it to the editor to decide whether this level of detail is appropriate given the purpose of the study or whether the text should be shortened again. It is important to note that while in the manuscript all feasibility measures are reported, we have focused on the most important feasibility measures in the abstract and therefore only describe those in the abstract methods.
Changes to the manuscript:
Abstract (page 2):
Acceptability: Acceptance and Feasibility Scale (AFS) and coach-rated engagement during coaching sessions. Demand: Number of completed computerized training, strategy coaching, and analogue game sessions. Implementation: E-Fit Fidelity Measurement System, assessing adherence to core components. Practicality: Retention rate. Integration: AFS integration items. Exploratory efficacy: Behavior Rating Inventory of Executive Function (BRIEF), neuropsychological EF testing and psychosocial variables at baseline, post-intervention (8 weeks), and at 4-month follow-up.

• Briefly report participant characteristics, including mean age, age range, and sex.
Response: Thank you for pointing this out. We have added this additional information.
Changes to the manuscript:
Abstract (page 2): Children 10 to 13 years (M=11.0, SD=0.9) with cCHD without a genetic diagnosis with infant open-heart surgery and reported difficulties (T-scores >60) on any of the summary scales of the parent- or teacher-reported Behavior Rating Inventory for Executive Function.
We recruited 42 participants (N_{female}=20).
• Specify whether analog games were played a median of nine times per week or in total.
Response: Thank you for this comment. We have now clarified that analogue games were played a median of nine times in total over the intervention period.
Changes to the manuscript:
Abstract (page 2): Demand: median number of computerised training sessions completed was 16 of 24 sessions (67%, (IQR; 6 to 19)), all children attended all scheduled coaching sessions, analogue games were played in total a median 9 times (IQR 4 to 13.25).
• Explain how practicality is measured by retention.
Response: Retention served as a marker of practicality, based on the assumption that an intervention that fits well within participants' available resources and

everyday contexts is more likely to be completed. This level of conceptual detail is beyond the scope of an abstract, but we have added a clarification to the Methods section explaining why retention was used as an indicator of practicality.

Changes to the manuscript:

2.3 Feasibility Measures (page 9): Retention rate served as an additional indicator of practicality, as higher retention suggests that the intervention fits within participants' available resources and everyday circumstances.

• Clarify the connection between social responsiveness and the intervention as an exploratory efficacy outcome.

Response: Thank you for this comment. Due to space limitations, we have described all secondary outcomes and the rationale behind their selection in the Supplementary Materials (Section 2: Rationale for Secondary Outcomes).

The text for Social Responsiveness Scale (SRS) is as follows:

The SRS is widely recognized as a valid measure for assessing social, communicative and rigid behaviour in children and adolescents in the sense of a dimensional diagnosis of autism. There is a high degree of convergence with autism spectrum disorders in the extreme range of the scale [24]. Research indicates that behavioral regulation processes, such as inhibition, shifting, and emotional control, are predictive of social functioning across both children with autism spectrum disorder and typically developing peers [25]. This suggests that deficits in EFs may contribute to social impairments, as individuals with lower EF skills often exhibit heightened difficulties in social interactions [25]. Additionally, interventions aimed at enhancing EFs have shown promise in improving social skills, suggesting a bidirectional relationship where better EFs can lead to improved social responsiveness [26,27]. Therefore, we decided to include the SRS as a secondary outcome measure.

• Outline the multimodal nature of the intervention and identify which modalities are used.

Response: Thank you for this comment. We have now explicitly outlined the multimodal nature of the intervention in the Abstract, specifying the three modalities included: (1) computerised EF training; (2) remote 1:1 individual EF strategy coaching; and (3) analogue games.

Changes to the manuscript:

Abstract (page 2): The 8-week intervention was multimodal including three modalities: 1) computerised EF training 3 x 20 min /week with CogniFit; 2) a weekly, remote standardised 1:1 individual EF strategy coaching; 3) analogue games played at convenience.

2. Introduction

The introduction paragraph provides a clear rationale for the study. In the review of prior studies, it would be helpful to specify how efficacy was measured—whether by parent report, clinician ratings, performance-based scores, or other methods—as the

list of studies suggests a mix of approaches. Clarification in this section would improve transparency.

Response: We agree with the reviewer that this is an important point and have clarified this accordingly.

Changes to the manuscript:

1 Introduction (page 4): EF intervention studies for children with cCHD are, to date, limited to three studies, two of which involve the computerised Cogmed© working memory training programme [31,32] and one using LEGO®-based therapy [33]. The first study using Cogmed© reported immediate postintervention improvements in performance-based working memory measures; however, these were not sustained longitudinally [32]. The second study, also using Cogmed©, found no improvement in working memory, although participants exhibited enhanced performance-based inhibitory control and parent-reported self-regulation at a 3-month follow-up [31]. The third study using the LEGO®-based therapy reported improvements in performance-based motor control, inhibitory control, risk selection, and total EFs at immediate post-intervention, but had no follow-up assessment [33]

At the end of page 5, the authors summarize literature supporting multimodal interventions. An additional sentence might explicitly clarify whether these interventions involve combining tasks targeting multiple executive function domains.

Response: Thank you for this comment. We have added this information to the respective studies and added a concluding statement at the end of the paragraph, to make the advantages clearer.

Changes to the manuscript:

1 Introduction (page 4-5):

In children with autism and in adolescents with epilepsy online individualized coaching sessions focussing on problem-solving strategies combined with computerised training targeting multiple EF domains improved EF behaviors [35,36]. Further, analogue games, such as board and card games targeting EFs, have improved EFs in children with ADHD. Their interactive nature may promote better self-monitoring in children [37,38]. Combining gamified computerised training paradigms with analogue games targeting reasoning and processing speed showed improvement in reasoning and speed tasks among healthy children from low socioeconomic backgrounds [39]. Within these multimodal interventions, also multiple EF domains could be targeted. These interventions enabled targeting multiple EF domains across modalities. Given that children with cCHD are at a higher risk to face challenges across multiple EF domains, a multimodal intervention approach appears particularly beneficial for enhancing their EFs.

On page six, line 5, the manuscript notes that the present study includes a comprehensive needs assessment. It may enhance clarity to rephrase this to indicate that the current study was informed by such an assessment, as the details are not presented within the manuscript aside from the review.

Response: Thank you for this helpful suggestion. We have revised the wording accordingly to clarify that the present study was informed by a comprehensive needs assessment, rather than including the assessment itself.

Changes to the manuscript:
1 Introduction (page 5): Hence, the present study was informed by a comprehensive needs assessment to incorporate the perspectives of patients, their families, teachers and healthcare professionals in the development of the intervention [40].

It would be useful to understand the authors' rationale for assessing feasibility alongside conducting a randomized controlled trial (RCT). Was the feasibility of E fit previously piloted before the RCT? If not, testing both the intervention's feasibility and its implementation within an RCT may introduce potential confounds.

Response: Thank you for this thoughtful comment. This issue was carefully discussed during the study design phase. We decided to combine the testing of the feasibility of E_Fit with an RCT. This decision was made because exploratory efficacy is considered a component of feasibility, as defined by Bowen and colleagues [1]. Assessing exploratory efficacy is important not only to inform the design of a future fully powered efficacy RCT, including sample size estimation, but also to evaluate whether the intervention shows sufficient promise to warrant continuation. Together with the other feasibility components, exploratory efficacy helps determine whether it is sensible to proceed with the current intervention or whether further refinement is required before advancing to a definitive trial. To assess exploratory efficacy appropriately and to account for potential developmental improvements in executive functions over time, the inclusion of a control group was therefore necessary.

We have not included this information in the introduction section of the manuscript for brevity reasons, but if requested would do so.

3. Methods & Analyses

Please clarify the rationale for stratifying by maternal education. Did the authors consider randomizing participants according to the severity of executive functioning concerns?

Response: Thank you for this very thoughtful question. Maternal education was used as a stratification variable as a proxy for socio-economic status, which has consistently been shown—both in this and in previous studies—to have a substantial impact on executive functioning. Given its strong influence, we considered this the most relevant variable for stratification.

Maternal education was chosen specifically because, in most cases, mothers completed the questionnaires. Although information on both parents' education was collected, we assumed maternal education to be the most reliable indicator in this context. Additionally, using a single parent's educational level avoids potential complications related to single-parent households, where information on the other parent's education may be incomplete or less reliable. Notably, this approach allowed us to retain complete data for all participants without the need for imputation.

We agree that stratifying by baseline executive functioning is an important consideration and discussed this during the study design phase. With a larger

sample size, this would indeed have been a strong candidate for an additional stratification variable. However, given the limited sample size of this feasibility study, we opted not to include it to avoid over-stratification. As baseline executive functioning is highly relevant both for intervention effects and adherence, we instead controlled for baseline executive functioning in the statistical analyses. We will, however, stratify for baseline executive functions in the currently planned full RCT.

How did participants respond to questions assessing feasibility? Was this interaction conducted with therapists, and if so, was it online? Is there any potential bias among therapists, particularly regarding the therapeutic relationship?

Response: Thank you for raising this important point. The feasibility measures were completed in different settings depending on the construct assessed. Ratings of fun, demand, and affect related to the computerized training were completed online by the participating children independently, potentially with a parent present. Coach-related ratings were completed at the end of the respective coaching sessions. In these cases, participants received the questionnaire link via the chat function and completed the ratings while still connected to the call. We acknowledge that this procedure may introduce potential bias related to the therapeutic relationship. To mitigate this, coaches explicitly reminded participants that they could not see the responses and encouraged them to answer honestly. The same procedure was applied for the ASF scale.

This approach was chosen to ensure complete and timely data collection in this feasibility study. The potential influence of therapist-related bias is therefore acknowledged as a limitation and will be considered when interpreting the results.

Changes to the manuscript:

Strengths and limitations of this study (page 3): L: Participants were unblinded following the baseline assessment, and some of the questionnaires were filled in in the presence of the investigators/coaches, potentially introducing a bias.

4.2 Strengths and limitations (page 16): Further, by responding to some of the questionnaires were in the presence of the investigators/coaches, potentially introduced a bias.

The authors are commended for their efforts in assessing possible contamination and acknowledging other activities that foster executive functioning. Could specific examples be provided of the computerized training methods and particular components of executive functioning targeted both within the program and during coaching sessions?

Response: Thank you for your appreciation and your comment. We have generally extended the description of the intervention and added some examples for each modality.

Changes to the manuscript:

2.2.1.2 Strategy coaching (page 6-7): The strategy coaching comprises eight sessions and each session is structured and standardized. The first session primarily serves as a feedback session, during which the child and parents are informed about

the results of the EFs assessment. As noted by Pletschko et al. [47], feedback of this kind already constitutes an element of neuropsychological intervention. Accordingly, the manual for the first session has been previously published and is taken from the book “Neuropsychologische Therapie mit Kindern und Jugendlichen” [47].
For the subsequent sessions, eleven potential EF topics are available, each linked to a task from the workbook Smart but Scattered by Dawson and Guare [46]. To identify the most relevant topics for each child, parents complete the corresponding questionnaire derived from the workbook. The six topics rated as most relevant are subsequently addressed in six strategy coaching sessions. Each of these sessions begins with a review of the previous week, followed by a brief story from Liebers et al., [48–51] and concludes with completion of the selected task from the workbook. Which story and which part of it specifically is tailored to the respective topic of the session. E.g. In one session, children would hear about a little dragon who is really scared of a maths test. First he can‘t seem to remember how to calculate the day before the test. But then his friend the rabbit comes a long and together they sing a song and breathe deeply. As the dragon calms down, he can remember everything and solve the last, preparing homework. Afterwards the child is asked to reflect in which situation they feel similarly, and what helps them in that situation. Together with the coach they then write down those situations, what they sometimes happen to do, and what they could try instead. The solutions are found along with the coach. Next time, a said situation arises, the child will try to use one of the tricks they’ve written down. This session aims to teach them emotion regulation and is either applied to the emotion fear or anger. After four sessions, parents are called to discuss upcoming sessions and to provide guidance on reinforcing EFs at home. The final session serves as a closing session and does not introduce any new topics. Instead, the preceding weeks are reviewed, with each session revisited to discuss the strategies applied as well as any achievements and remaining difficulties. Families are encouraged to continue practicing the strategies that have proven effective for them.

The authors refer to a manual standardizing the coaching sessions, which appears to be Dawson and Guare’s executive functioning workbook (Smart but Scattered). How was this resource leveraged and adapted for the study?

Response: Thank you for this valuable comment. We agree that a more detailed description of the manual and its sources is important. Accordingly, we have revised the manuscript to provide a more comprehensive explanation of the manual, explicitly referencing the relevant sources and clarifying how they were adapted and applied within the intervention. See the adjustments above.

Were coaching sessions recorded for coding or fidelity assessment purposes?

Response:

Thank you for pointing this out. Indeed, the coaching sessions were recorded to assess intervention fidelity. This hasn’t been properly described yet, we have

therefore revised the manuscript accordingly and added a sentence to explicitly describe this procedure.

Changes to the manuscript:

2.3 Feasibility measures (page 9): [...] It evaluated how closely all the E-Fit components were implemented, including core components, specific questions, and session completions, with yes/no response options. For this purpose, the strategy coaching sessions had been recorded.

While it is acknowledged that efficacy measures are exploratory, I recommend narrowing the range of outcomes analyzed and directly linking them to executive functioning domains addressed by the interventions. If the intervention is personalized, changes would only be expected in targeted domains, making analysis complex. Consider focusing on core or primary outcomes, as the current sample size may not support the number of variables included.

Response: We are very grateful for this insightful comment. We found it challenging to determine the appropriate focus for this paper, and this suggestion was particularly helpful. As all three modalities tap into cognitive flexibility, working memory, planning, and inhibition, we selected one representative measure for each of these domains. The manuscript has been revised accordingly.

Changes to the manuscript:

2.3 Measures (page 9-10): Exploratory efficacy was investigated through baseline, post-intervention, and 4-month follow-up assessments of everyday EFs using the BRIEF [43] and neuropsychological EF performance. All three intervention modalities tapped into the four EF domains including flexibility, working memory, planning and inhibition. To get an exploratory idea about potential efficacy, we have therefore looked at one assessment for each of those domains:

Flexibility: Delis-Kaplan Executive Function System (D-KEFS) Trail Making Test [58];

Working memory: Wechsler Intelligence Scale for Children - 5th edition (WISC-V): Digit Span [59]

Planning: D-KEFS Tower Task [58];

Inhibition: D-KEFS Color Word Interference Test [58]; Analyses were conducted with raw scores for all measures.

3.2.1 Primary outcomes: Feasibility (page 13): *Exploratory efficacy*: *For the neurodevelopmental assessment, only negligible effect sizes were observed in the Trail-Making Test as a measure of flexibility, the Digit span as a measure for working memory and the CWIT as a measure of inhibition. A small effect was observed in the Tower Task at T2. Notably, small and moderate effects were observed in the BRIEF GEC parental report. This was particularly driven by* the Behavioral Regulation Index (BRI) of the BRIEF parent-report showing a small effect size for the post intervention and a large effect size for the 4-month follow-up, both favouring the intervention group (table 2 and figure 4). The detailed results at post-intervention are presented in Supplementary table 3 and at 4-month follow-up in Supplementary table 4.

Under descriptive measures, please clarify the protocol followed in cases where only one source of education was reported, or in single-parent households.
Response: Thank you for highlighting this important point, which had not been addressed in the manuscript previously. We have now included a description of the procedure used in the Methods section.
Changes to the manuscript:
2.4 Descriptive measures (page 10): The questionnaire collecting information on parental education was completed by one parent for both parents, regardless of whether there was a single-parent household. Missing values were imputed using multivariate imputation by chained equations.

A notable strength of the analysis is the capacity to observe trends and within-subject ratings across the intervention period.
Please specify how coaching sessions were rated—were assessments based on video recordings or did multiple evaluators participate in sessions?
Response:
We thank the reviewer for raising this point. The sessions were recorded, and rated by multiple evaluators who did not participate in the sessions. We have clarified this in the manuscript, both in the referenced section and in the Methods section.
Changes to the manuscript:
2.3 Feasibility measures (page 9): Practicality was evaluated using practicality targeted questions from the AFS (e.g. "Everything worked as it should" or "It was easy to use the online website for the games"), observed coach involvement during coaching sessions (rating sheet, separately rated by two separate raters, 1 to 3 scale [54], based on strategy coaching recordings), and the Session Rating Scale after each coaching session (rated by the participants, 0 to 10 scale [57]).

The statistical approach is appropriate; however, the study appears underpowered for the number of comparisons made. As noted above, I recommend prioritizing and possibly grouping outcome types to ensure analyses are clearly tied to intervention goals and rationales. Currently, outcome measures seem broadly associated with executive functioning without a consistent connection to the intervention itself.
Response: Thank you again for this important point. We have revised the manuscript accordingly, as noted above, and believe that these changes substantially improve the readability and clarity of the paper.

4. Results
The consent rate is 24%.
Response: Thank you for pointing this out. We agree that differentiating the absolute numbers and recruitment percentages improves clarity. Accordingly, we have revised the wording in Table 1 to clearly distinguish between these values.
Changes to the manuscript:

See *Table 2. Feasibility: areas of focus, assessment tools, results and interpretation* (pages 19-21).

There are several "error reference not found" notices in the results section, beginning at the top of page 11. There is also inconsistent capitalization, for example, in the tables.
Response: Thank you for this remark. We apologize for this oversight and have corrected the erroneous citations accordingly.

The authors mention qualitative analysis of participant feedback, but the specific qualitative analytical approach is not described in the analysis section.
Response: Thank you for this comment! We have added more details about this analysis to the manuscript.
Changes to the manuscript:
2.5 Statistical analysis (page 10-11): Two raters independently evaluated coaching sessions using the E-Fit Fidelity Measurement System, and inter-rater reliability was reported. Any qualitative data, such as reasons for nonparticipation and feedback within coaching sessions, were analysed and reported with structured qualitative content analysis [64]. <u>Initially, main categories were derived inductively from the data. Subsequently, the material was systematically coded. The coding process was iterative, allowing for refinement of the category system. All data were then coded using the final category system, enabling structured comparison and interpretation of relevant content across cases.</u>

Retention was high in some aspects of the program; however, only four children completed 80% of the computerized training, which may indicate limited fidelity or acceptability for this modality.
Response: Thank you for this valuable suggestion. We have incorporated this point into the Discussion to ensure readers are aware of this potential bias.
Changes to the manuscript:
4.1 Feasibility outcomes (page 14): However, adherence to the computerised training schedule was inconsistent. A technical issue with the CogniFit software limited session duration to 10 minutes instead of the intended 20 minutes, requiring children to complete two consecutive sessions- a requirement not always fulfilled. <u>The limited adherence to this modality may also restrict the interpretability of the feasibility findings related to the computerized training, as these assessments were predominantly completed by participants who engaged in the training.</u>

Regarding the number of analyses conducted for the exploratory results, the presentation in the results section could be improved. As previously suggested, focusing on two to three key outcome variables and presenting their effect sizes in the results would strengthen the section. The manuscript refers too frequently to supplementary tables; it is recommended to identify core tables for inclusion in the main results section and to report relevant statistics.

Response:
Thank you again for this important point. We have revised the manuscript accordingly, as noted above, and believe that these changes substantially improve the readability and clarity of the paper. We now present key outcome variables for exploratory efficacy in the main manuscript, namely flexibility (Trail Making Test flexibility condition speed), working memory (Digit span number correct), planning (Planning Tower Task achievement score), inhibition (Color Word Interference Test inhibition condition speed) and BRIEF parents and teacher Global Executive Composite scores We have added them to the Table 2, along with the other feasibility results.

5. Discussion
The authors introduce a creative and original intervention; however, it remains unclear at this stage how the approach was specifically tailored to the needs of patients with CHD, how it might differ for individuals with other medical conditions, or whether the customization is solely based on the unique executive functioning challenges of each child.
Response:
Thank you for this insightful question. The intervention was developed specifically for children with cCHD, who, at the group level, are known to have an increased risk of difficulties across multiple domains of executive functioning. For this reason, the program was designed to address a broad range of executive functions rather than focusing on a single domain.
In addition, the intervention was refined through the active involvement of patients and their parents, allowing us to align the content with their needs and preferences. This included topics like the ideal age, the wish for direct strategy coaching and an analog component to the intervention. In this sense, the intervention is tailored to the cCHD population. At the same time, recognizing the substantial heterogeneity within this group, the program allows for further individualization to accommodate each child's unique profile.
While the intervention was developed for children with cCHD, similar executive function difficulties are observed in other pediatric populations, such as children born preterm. At the same time, the needs and preferences expressed by patients and parents largely reflected those of children with executive function difficulties in general.
Therefore, the intervention may also be applicable to broader populations characterized by diffuse executive function difficulties who are largely able to attend regular education.
We have added this aspect in the conclusion section of the discussion.
Changes to the manuscript:
4.3 Conclusions (page 16)
While the E-Fit intervention was developed specifically for children with cCHD through the active involvement of patients and their parents, allowing us to align the content with their needs and preferences. similar EF difficulties can be observed in

other pediatric populations, such as children born preterm [84]. At the same time, the needs and preferences expressed by patients and parents largely reflected those of children with executive function difficulties in general.

It is challenging to reconcile reports describing the cognitive training component as both enjoyable and demanding with the observed low rate of completion among children in this segment. Reference is made to the small number of participants who completed at least 80% of these sessions.

Response: Thank you again for this important point. Although only a few children completed more than 80% of the suggested sessions, all participants completed these questionnaires. As they rated them as enjoyable but at the same time as demanding, indicates that the vames remained challenging throughout the training. Nevertheless, we agree that the interpretation of these results requires caution. We have therefore revised the manuscript accordingly, as outlined above. We believe this information remains important and hope that the revised text explains these considerations clearly to the reader.

The discussion could be strengthened by providing a more coherent and concise summary of the results section, particularly after further revisions to that section are made. Currently, some results referenced in the discussion are only found within supplementary analyses sections.

Response: Thank you for this helpful comment. In response, we have adjusted the Results section and ensured that all key findings are now reported in the main manuscript, improving their accessibility. We have also revised the Discussion section accordingly (see below) to provide a more coherent and concise summary of these results and to ensure that all findings discussed are clearly presented in the main text.

Changes to the manuscript:

4.1 Feasibility outcomes (page 15):

Exploratory efficacy analyses showed promising results, particularly in parent-reported EFs showing a large effect size for behavioral regulation and a moderate effect size for the general EF scores. This effect was not only detected directly post-intervention, but also at the 4-month follow-up. In the neuropsychologically assessed EFs, only small effects could be found for planning at T2. Thus, the intervention effects were primarily evident in everyday functioning rather than in standardized test performance. The strategy coaching, which showed the highest adherence and therefore the greatest intervention dose, specifically targeted strategies aimed at improving daily functioning. With increased adherence to the other intervention modalities—and consequently a higher overall dose—these effects may eventually also be reflected in standardized test outcomes.

While the study offers a comprehensive overview of its limitations, the specific challenges identified among children and families from lower SES backgrounds remain unclear, as these were not reflected in the results. Generally, there appeared

to be difficulties engaging children in certain aspects of the intervention, but a direct connection to SES was not evident. If SES is indeed a factor, this should be substantiated within the results section. The only significant differences highlighted pertained to surgeries involving cardiopulmonary bypass and participation in sports activities.

Response: Thank you for this comment. Although the mean SES was reported in Table 1, we had not previously clarified how this value should be interpreted. While the present study did not specifically examine the challenges faced by children from lower SES backgrounds, these challenges have been well documented in previous research focusing on group effects and using SES as a covariate. We have now added a clarification in the Discussion section. We hope that these additions make it clearer to the reader why reaching children from lower SES backgrounds is particularly important for this type of intervention.

Changes to the manuscript:

4.2 Strengths and limitations (page 15-16):

SES among participants was relatively high, also compared to the general population in Switzerland [89]. Previous studies have shown, that lower SES is a risk factor for reduced EFs in children with cCHD [90], even more than perioperative total brain volume or brain injury severity.

The authors are advised to moderate their conclusions regarding the efficacy of the intervention. It may be beneficial to establish a priori feasibility metrics to confidently assert the feasibility of the intervention period; at present, concerns regarding adherence and participation persist and warrant cautious interpretation.

Response: Thank you for this thoughtful suggestion. We agree that the conclusions regarding intervention efficacy should be interpreted cautiously. Accordingly, we have moderated the wording of the conclusions and emphasized the feasibility focus of the study. In addition, we described the observed challenges related to adherence and participation (see above). These points are now more explicitly addressed in the revised manuscript.

Changes to the manuscript:

4.1 Feasibility outcomes (page 15): Exploratory efficacy analyses showed promising results, particularly in parent-reported EFs showing a large effect size for behavioral regulation and a moderate effect size for the general EF scores. This effect was not only detected directly post-intervention, but also at the 4-month follow-up. In the neuropsychologically assessed EFs only small effects could be found for planning at T2. Thus, the intervention effects were primarily evident in everyday functioning rather than in standardized test performance. The strategy coaching, which showed the highest adherence and therefore the greatest intervention dose, specifically targeted strategies aimed at improving daily functioning. With increased adherence to the other intervention modalities—and consequently a higher overall dose—these effects may eventually also be reflected in standardized test outcomes.

6. Tables:

Table 1
The brief GEC parent report difference between groups is concerning; future randomization should address this through stratified methods.
Response: Thank you for this valuable suggestion. We agree that the observed between-group difference in the BRIEF GEC parent report at baseline warrants attention, and we will address this in the planned randomized controlled trial by considering stratified randomization procedures to better balance groups on key baseline characteristics.

Table 2 is useful, but the results should be more synthesized and clearer when presenting percentiles, means, or medians. A formal definition of "supportive" and "non-supportive" would also strengthen the authors' interpretation.
Response:
We thank the reviewer for this comment. As suggested by you, we have changed the section “exploratory efficacy” of the Table 2 to report targeted EF and present now also percentiles, means or medians also in that section.
Regarding the definition of “supportive” and “non-supportive”, we would like to point out that this definition has been provided in the notes below the Table 2. Specifically, we defined supportive = supports the intervention group. A result was rated as supportive if it met the following thresholds: for outcomes rated on a 1 to 4 scale, a median rating of ≥3 was considered supportive; for outcomes rated on a 1 to 3 scale, a median rating of 3 was considered supportive; for outcomes rated on a -5 to 5 scale, a median rating of ≥ 1 was considered supportive; for outcomes rated on a 0 to 10 scale, a median rating of ≥6 was considered supportive. For the recruitment rate, a threshold of 75% and for the retention rate, a threshold of 90% were defined as supportive. Effect sizes were defined using adjusted Hedge’s gA1: small effects (≥ 0.2 and < 0.5) were rated as partly supportive, and moderate or larger effects (≥ 0.5) as supportive.

Separate the primary feasibility outcomes into a single table. The current presentation of exploratory efficacy variables is too vague; it’s not appropriate to group entire measures like WISC or DKEFS together. Specify the number of analyses, variables, and relevant indices for each subtest.
Response: Thank you again for this important point. We have revised the manuscript accordingly, as noted above, and believe that these changes substantially improve the readability and clarity of the paper.

7. Figures:

Figure 1
Can the author explain why so many were unreachable? Is this common in their clinical follow-up for congenital heart disease?
Response:

We agree with the reviewer that the number of patients who could not be reached is high. This is in part explained as about two thirds of patients who have been operated in our tertiary hospital will have their subsequent cardiac and general pediatric follow-up assessments in specialized regional cardiac clinics. Thus, patient contact information may not be valid anymore. Despite the efforts by the study team to trace potential participants, including contacting their former pediatricians, valid contact information could not be identified for many individuals. Others did not respond to either the invitation letter or follow-up phone calls; the reasons for non-response are unknown but may include language barriers or difficulties understanding the study information. To address this, we are currently revising and improving the recruitment materials.

What is the purpose of Figure 4? It includes data for four participants?
Response: Thank you for this comment. We identified a mix-up in the figures and have corrected it accordingly. The manuscript includes three figures; the issue raised by the reviewer appears to refer to Figure 2. We have now added revised the figure note to improve clarity and facilitate interpretation for readers and hope it will resolve this comment.
Changes to the manuscript:

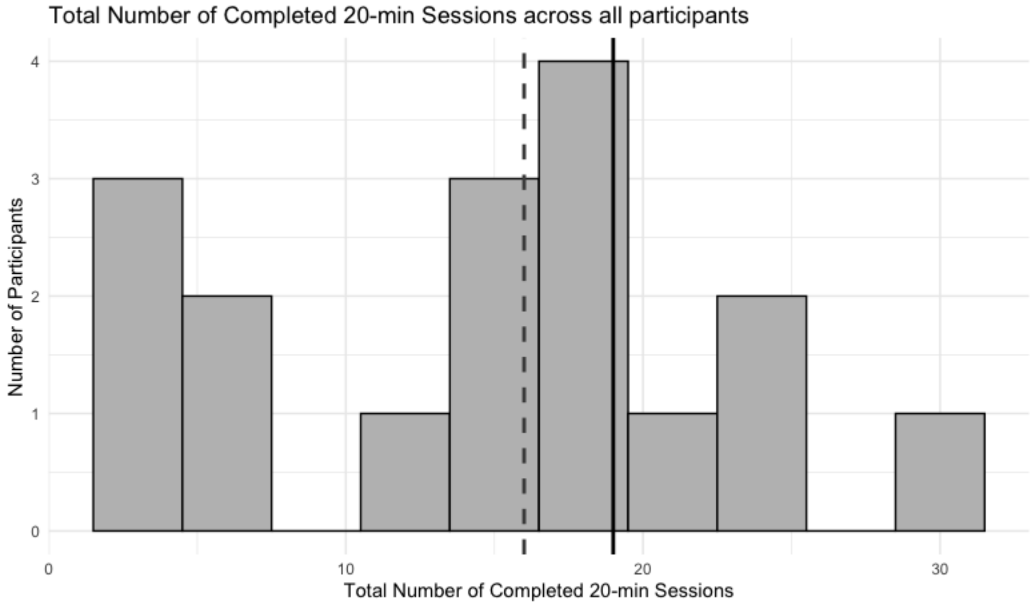

Figure 2. Distribution of the total number of completed 20-minute sessions per participant. Bars represent the number of participants within each session range. The dashed vertical line indicates the median number of completed sessions (median = 16.0, IQR = 6.0 to 19.0), and the solid vertical line indicates the 80% training completion threshold (~20).

Page 27 lacks clarity regarding whether it references an appendix or a justification of measures. There should be a clear presentation of stratified outcomes and screening tools.
Response: Thank you for this comment. We believe this confusion may have arisen due to the format in which the document was reviewed. The reviewer likely refers to page 2 of the supplementary material (Section 2: Rationale for Secondary Outcomes). As this section is part of the supplementary, it is intended to provide

additional background information, specifically the rationale underlying the selection of the secondary outcome measures. The description and presentation of these measures are provided in the main manuscript in Section 2.3 (Feasibility Measures, page 9). We hope that this clarification resolves the issue and are, of course, open to further suggestions if any ambiguity remains.

The qualitative analysis appears descriptive; was the interview data synthesized in terms of percentages of families reporting opportunities across indices? Please clarify.

Response: Thank you for this question. The qualitative analysis was intentionally descriptive, and interview data were not quantified or synthesized using percentages across indices. We have added an explanatory sentence to the supplementary material.

Changes to the manuscript:

Supplementary 2: Qualitative analysis (page 3): The following descriptive synthesis presents feedback from the coaching sessions summarized into indices; it is not intended to be quantified and should not be interpreted as such.

It's unclear how "calm activities" differ from EF-enhancing activities if they aid behaviour regulation.

Response: Thank you for this helpful comment. We agree that the rationale underlying this distinction was not sufficiently clear in the original text. The categorization was based on the specific activities reported in free text and summarized in Supplementary Table 2. While these "calm activities" may indeed support behavioral regulation, they were conceptually distinct from the EF-enhancing activities explicitly trained in the intervention. Importantly, assessing potential regulatory effects of these activities was beyond the scope of the present study. The primary purpose of the activity log data was to document activities undertaken outside the intervention and to ensure that the control group did not engage in activities equivalent to those provided to the intervention group, which could occur given that the intervention protocol was published and participants were not blinded to group allocation. We have clarified this distinction in the manuscript.

Changes to the manuscript:

2.2.2 Control group (page 8):

To track and monitor the duration of activities that could enhance EFs, parents and their children were asked to maintain an electronic activity log four times a week for 8 weeks. In the log, participants selected their activities for each 15-minute interval throughout the day from a drop-down menu <u>(sleeping, eating, school lessons, physical education, break time, homework, reading, playing an instrument or singing, watching television, computer games, quiet activities, indoor exercise, outdoor exercise, vigorous indoor exercise, vigorous outdoor exercise, indoor training (in a club), outdoor training (in a club), walking, cycling, public transport, car, other activities). For further specification within these categories, families filled in free text, what those included at the end (see Supplementary Table 2).</u>

Supplemental Table 3 lists about 16 neuropsychological test outcomes but omits supplementary questionnaire variables shown in Supplemental Table 1.
Response: We agree that Supplementary Table 3 did not include the questionnaire variables. These variables are presented in separate tables (Supplementary Tables 5 and 6). Following the restructuring of the Results section and the revision of Table 2 in the main manuscript, Supplementary Tables 3 and 4 as well as the tables including the questionnaire-based outcomes remain. We thank the reviewer for the helpful suggestions, which contributed to a clearer presentation of the results.

Reviewer: 2
Dr. Anne Gallagher, Université de Montréal
Comments to the Author:
This is a very well-written manuscript and an extremely important and relevant contribution given the frequent executive function challenges in children with CHD and their impact on daily life and quality of life. My comments and suggestions are generally minor but hopefully will help to clarify certain points.

Abstract
In the Interventions section, it is not clear whether there are 2, 3, or 4 groups. This point should be clarified. In Section 2.2.1, it is very clear that the study included 2 groups.
Response: Thank you for this important comment. We have clarified in the Interventions section that the study included two groups, consistent with the description in Section 2.2.1, and revised the text accordingly to avoid any ambiguity.
Changes to the manuscript:
Abstract (page 2): Interventions: Children with cCHD were randomly assigned to <u>one of two groups</u>: the intervention or the control group.
Methods and Results
Why was the randomization stratified by maternal education? This is interesting.
Response: Thank you for this very good and thoughtful question. Maternal education was used as a stratification variable as a proxy for socio-economic status, which has consistently been shown—both in this and in previous studies—to have a substantial impact on executive functioning. Given its strong influence, we considered this the most relevant variable for stratification.
Maternal education was chosen specifically because, in most cases, mothers completed the questionnaires. Although information on both parents' education was collected, we assumed maternal education to be the most reliable indicator in this context. Additionally, using a single parent's educational level avoids potential complications related to single-parent households, where information on the other parent's education may be incomplete or less reliable.

Changes to the manuscript:

2.1 Study design and population (page 6):
How was the 4-month duration for the long-term follow-up determined? I recommend justifying this in the manuscript. Is there evidence that 4 months post-intervention is sufficient to demonstrate a long-term or lasting effect of the intervention?
Response: Thank you for this remark. We agree that a justification makes sense and added a sentence to the manuscript, along with references.
Changes to the manuscript:
1 Introduction (page 6): <u>The 4-month follow-up was selected to be consistent with previous studies examining longer-term effects, in which such effects were variably observed [31,32].</u>

How were the activities presented in Supplementary Table 2 selected?
Response: The general activity categories presented in Supplementary Figure 2 were derived from a previously used and published activity log for Swiss school-aged children. However, as these categories were relatively broad, families were additionally asked to specify the exact activities using free-text entries. For example, while children indicated the number of minutes per day spent reading or listening to audiobooks, the type of media consumed within this time may vary substantially. Therefore, families provided details on the specific books and audiobooks their child was reading or listening to. These free-text entries are summarized in Supplementary Table 2. The relationship between Supplementary Figure 2 and Supplementary Table 2 has been clarified in the supplementary text.
2.2.2 Control group (page 8):
To track and monitor the duration of activities that could enhance EFs, parents and their children were asked to maintain an electronic activity log four times a week for 8 weeks. In the log, participants selected their activities for each 15-minute interval throughout the day from a drop-down menu <u>(sleeping, eating, school lessons, physical education, break time, homework, reading, playing an instrument or singing, watching television, computer games, quiet activities, indoor exercise, outdoor exercise, vigorous indoor exercise, vigorous outdoor exercise, indoor training (in a club), outdoor training (in a club), walking, cycling, public transport, car, other activities). For further specification within these categories, families filled in free text, what those included at the end (see Supplementary Table 2).</u>

It is mentioned for several measures that they were evaluated "based on integration targeted questions from the AFS". For readers who are not familiar with this scale (myself included), it would be helpful to provide one or more examples of the AFS questions used each time this is mentioned.
Response: Thank you for this important suggestion. We agree that this would help readers better understand the areas of focus, as well as the results and have added examples to the manuscript.
Changes to the manuscript:
2.3 Feasibility measures (page 8-9):

Acceptability was evaluated with acceptability targeted questions from the Acceptance and Feasibility Scale (AFS [35]). Although labeled 'acceptance and feasibility scale,' these questions covered multiple areas of focus as defined by Bowen et al. [48]. To reflect this, we grouped the questions accordingly. It also featured exploratory items allowing participants to indicate which aspects they found most helpful, most enjoyable, or least relevant [35]. Acceptability-targeted questions included questions such as "I liked the online games" or "I felt that the coach contributed to my learning beyond the sessions". […]
Demand was measured with the demand targeted questions from the AFS (e.g. "I would do the programme (as a whole) again" or "I would recommend the programme (as a whole) to other children"), as were children's ratings of how fun and cognitively demanding the computerised training was (Fun and Demand, 1 to 5 scale [51]). […]
Practicality was evaluated using practicality targeted questions from the AFS (e.g. "Everything worked as it should" or "It was easy to use the online website for the games"), observed coach involvement during coaching sessions (rating sheet, separately rated by two separate raters, 1 to 3 scale [50], based on strategy coaching recordings), and the Session Rating Scale after each coaching session (rated by the participants, 0 to 10 scale [53]). […]
Integration was assessed based on integration targeted questions from the AFS such as "A face-to-face meeting with a coach would be better" or "the programme (as a whole) was too long".

"Analyses were conducted with raw scores for all measures." Why were raw scores used instead of standard scores? How is the effect of age accounted for when using raw scores? Was age controlled for in the analyses?
Response: Thank you for this thoughtful question. We carefully considered whether to use raw or standardized scores and ultimately opted for raw scores because age-normed standard scores introduced interpretational challenges in this longitudinal design. Specifically, for some measures (e.g., the BRIEF), a child could show genuine improvement or deterioration over time but cross an age boundary between assessment time points (e.g., due to a birthday). This could result in an apparent change in standardized scores that reflects a shift to an older normative reference group rather than true change in executive functioning. Using raw scores allowed us to more directly capture within-subject change over time. Importantly, age was included as a covariate in all relevant analyses to account for age-related effects.

I am not sure about all the measures, but repeating certain WISC-V subtests after 4 months will almost certainly introduce a practice effect. What is the potential impact of this on the results?
Response: Thank you for this insightful comment. We agree that repeating WISC-V subtests within intervals of 2 and/or 4 months is not standard practice in clinical settings and may introduce practice effects, potentially leading to an overestimation of performance at subsequent time points. However, for the purposes of this study, follow-up assessments were not interpreted as stand-alone measures. Instead, they

were evaluated relative to each participant's baseline performance to assess intraindividual change over time, with these changes subsequently compared between the intervention and control groups. Because the control group underwent the same assessments at identical time intervals, any practice effects would be expected to occur in both groups and are therefore accounted for in the present analyses. We nevertheless acknowledge that follow-up scores should not be interpreted outside the specific context of this study.

Discussion

In the first paragraph of the Discussion, the authors state: "...suggest EF improvements particularly in behavioral regulation and effect on social responsiveness." It is not clear to me which specific results support this claim.

Response: Thank you for this comment. Throughout the revision process, we received very helpful feedback on how to present the results more clearly and to focus on the most relevant findings to enhance readability. In response, we revised the results section accordingly and moved key results from the supplementary material into the main manuscript to ensure they are more easily accessible to the reader.

Changes to the manuscript:

3.2.1 Primary outcomes: Feasibility (page 13): *Exploratory efficacy*: For the neurodevelopmental assessment, only negligible effect sizes were observed in the Trail-Making Test as a measure of flexibility, the Digit span as a measure for working memory and the CWIT as a measure of inhibition. A small effect was observed in the Tower Task at T2. Notably, small and moderate effects were observed in the BRIEF GEC parental report. This was particularly driven by the Behavioral Regulation Index (BRI) of the BRIEF parent-report showing a small effect size for the post intervention and a large effect size for the 4-month follow-up, both favouring the intervention group (table 2 and figure 4). The detailed results at post-intervention are presented in Supplementary table 3 and at 4-month follow-up in Supplementary table 4.

Few technical issues are reported in the manuscript. Will these technical issues be addressed in the next version of the intervention?

Response: Thank you for this important question. Yes, the reported technical issues are being addressed in the next iteration of the intervention. We are currently working on improving the accessibility and usability of the computerized training. In addition, an additional feasibility phase has been conducted using an alternative online platform for the strategy coaching component, allowing us to identify and select the most suitable platform for the final version of the intervention.

The exploratory analyses for EF improvements between BL and T1 and T2 (Suppl. Tables 3 and 4) are interesting. Although improvements appear greater in the Intervention group, some improvements are also present in the Control group for certain measures. Are the effect sizes reported only for the Intervention group? Did the authors compare the magnitude of improvement in the intervention group versus

the control group?
Response: Thank you for this very good observation. The reported effect sizes reflect the between-group differences in change over time, that is, they represent the magnitude of improvement in the intervention group relative to the control group. Although some improvements were observed in the control group for certain measures, the effect sizes quantify the differential change between groups rather than within-group change in the intervention group alone.

What are the next steps? Another feasibility study to improve recruitment rate, address technical issues, etc.? Or a transition to a full RCT? Did this feasibility study aim to establish the sample size for a future full RCT?
Response: We are very excited to take this intervention to the next phase. We are currently conducting an additional feasibility loop to incorporate adjustments aimed at improving adherence to the computerized training and engagement in the coaching sessions. In parallel, we are developing a more child-friendly implementation of the computerized training and revising the recruitment materials based on patient-informed needs assessments. Funding for a full randomized controlled trial has already been secured, and we plan to integrate these refinements and the present findings into the trial design, with recruitment anticipated to begin in the first half of 2026. Based on the results of this feasibility study, we were also able to perform a sample size calculation for the forthcoming RCT.

Minor comments :
- Pages 9, 10 and 11 "Error! Reference source not found" – please add the references (3)
Response: Thank you for pointing this out, and we apologize for the oversight. The missing references on pages 9, 10, and 11 have now been corrected accordingly.

- Page 12: "The CogniFit training component was perceived as both fun and cognitively demanding, which is an ideal combination for EF improvement. " Please provide a reference.
Response: Thank you for this remark. We agree that a reference was required and have now added the appropriate citation.

- Supplementary Table.2 There are some typos and German words.
Response: Thank you for this comment. Some games, television series, and books do not exist in English; therefore, we chose not to translate their original titles in order to allow proper identification.

- Page 13, line 2: replace . by ,
Response: Thank you for the rigorous reading of our article. We couldn't clearly identify this exact location, but have double-checked the surrounding pages for punctuation.

VERSION 2 - REVIEW

**Reviewer**	**1**
**Name**	**Williams, Tricia**
**Affiliation**	**The Hospital for Sick Children**
**Date**	**06-Feb-2026**
**COI**	

Thank you for the opportunity to review this the revised manuscript. I appreciate the efforts made to address previous feedback and improve the clarity and organization of the paper. Below, I have structured my comments to aid further refinement and ensure the manuscript meets publication standards.

Methodological Clarifications

• Qualitative Analyses: Please specify who completed the qualitative analyses. Clear identification of the analysts will enhance transparency and credibility.

• Group Comparisons: Clarify whether groups differed by descriptive variables such as gender, cCHD severity, or other relevant characteristics. Providing this information will help contextualize the findings.

Writing and Organization

• Brevity and Repetitiveness: The general writing would benefit from further review for brevity and avoidance of repetitiveness. Streamlining the text will improve readability and focus.

• Aims of the Study: There is no need to repeat the aims of the study in the results section. Consider removing redundant statements to maintain conciseness.

Results and Interpretation

• Moderate Engagement: Please clarify what is meant by "moderate engagement." Defining this term will help readers understand the relevance and implications of the results.

• Outcome Measures and Tables: As recommended in the prior review, consider abbreviating the number of outcome measures and reducing the number of supplementary tables. This will help focus attention on the most significant findings.

• Experimental vs. Control Group: It remains unclear from the efficacy analyses whether changes in the experimental group differ from any changes in the control group. Please clarify this point to strengthen the interpretation of results.

Development and Customization

• cCHD-Specific Development: The additional clarification regarding development specific for cCHD is appreciated. However, it would be helpful to elaborate on how the intervention was customized. Were there any discussions with the coach regarding lived experience? Providing this detail will enhance the manuscript's depth and relevance.

Summary

Overall, the manuscript demonstrates progress and addresses several points raised previously. Addressing the above specific suggestions will further strengthen the paper and its contribution to the field. Thank you for your continued work on this important topic.

**Reviewer**	**2**
**Name**	**Gallagher, Anne**
**Affiliation**	**Université de Montréal**
**Date**	**05-Feb-2026**
**COI**	

The authors have effectively addressed my comments and concerns and should be commended for their efforts. I therefore recommend this manuscript for publication.

VERSION 2 - AUTHOR RESPONSE

Reviewer #1:
Dr. Tricia Williams, The Hospital for Sick Children
Comments to the Author:
Thank you for the opportunity to review this the revised manuscript. I appreciate the efforts made to address previous feedback and improve the clarity and organization of the paper. Below, I have structured my comments to aid further refinement and ensure the manuscript meets publication standards.

Methodological Clarifications

Qualitative Analyses: Please specify who completed the qualitative analyses. Clear identification of the analysts will enhance transparency and credibility.

Response: We thank the reviewer for this valuable suggestion. We have clarified that the qualitative analyses were conducted by the first author (AS).

Changes to the manuscript:

Supplementary 2: Qualitative analysis (page 3): The following descriptive synthesis presents feedback from the coaching sessions summarized into categories; it is not intended to be quantified and should not be interpreted as such. The qualitative analyses were conducted by the first author (AS), following established procedures for structured qualitative content analysis.

Group Comparisons: Clarify whether groups differed by descriptive variables such as gender, cCHD severity, or other relevant characteristics. Providing this information will help contextualize the findings.

Response: Thank you for this comment. Group differences in descriptive baseline variables (e.g., gender distribution, cCHD severity, and other clinical characteristics) are reported in table 1 and described in the Results section (page 12, paragraph 3.1). For clarity, we have now added an explicit sentence in the paragraph presenting the group comparisons referring readers to Table 1.

Changes to the manuscript:
3.2.1 Primary outcome: Feasibility (page 13):
Exploratory efficacy: Baseline descriptive characteristics of the groups are presented in table 1. For the neurodevelopmental assessment, only negligible effect sizes were observed in the Trail-Making Test as a measure of flexibility, […]

Writing and Organization

Brevity and Repetitiveness: The general writing would benefit from further review for brevity and avoidance of repetitiveness. Streamlining the text will improve readability and focus.

Response: We thank the reviewer for this helpful comment. We agree that brevity and clarity are important for readability. We have carefully reviewed the manuscript and streamlined the text throughout. Redundant sections have been removed and several sections have been revised to reduce repetitiveness and improve focus.

Aims of the Study: There is no need to repeat the aims of the study in the results section. Consider removing redundant statements to maintain conciseness.

Response: Thank you for this suggestion. We agree and have removed the paragraph repeating the aims at the beginning of the results section.

Results and Interpretation

Moderate Engagement: Please clarify what is meant by "moderate engagement." Defining this term will help readers understand the relevance and implications of the results.

Response: Thank you for this important note. We agree, that this measure is not entirely clear and have clarified the meanings of the different stages of engagement in the methods section.

Changes to the manuscript:
2.3 Feasibility measures (page 9):
[…] Coaches rated participants' observed engagement on a rating sheet during the coaching sessions (low, moderate or high [54]). Low engagement reflected predominantly passive, distracted, and affectively flat behavior, whereas high engagement reflected focused, effortful, verbally active, persistent, and emotionally positive behavior. Moderate engagement represented mean ratings in the mid-range across these same dimensions, indicating neither predominantly disengaged nor highly engaged behavior. […]

Outcome Measures and Tables: As recommended in the prior review, consider abbreviating the number of outcome measures and reducing the number of supplementary tables. This will help focus attention on the most significant findings.

Response: We appreciate this suggestion and have further reduced the number of reported outcomes to strengthen the focus of the manuscript. The main text now emphasizes the core outcomes as defined in the previous review, and the supplementary material has been condensed from four to two tables: any tests not referenced in the revised manuscript have been removed from the supplementary tables and secondary and primary outcomes are now presented in the same tables. While this required removing additional results, we believe this streamlined presentation improves clarity and highlights the most relevant findings.

Changes to the manuscript:

Supplementary (page 6-7): Supplementary table 3 and 4

Experimental vs. Control Group: It remains unclear from the efficacy analyses whether changes in the experimental group differ from any changes in the control group. Please clarify this point to strengthen the interpretation of results.

Response: Thank you for this helpful comment. We have revised the Methods section to clarify that the ANCOVA models explicitly tested whether the intervention group showed greater improvement than the control group while adjusting for baseline values. Specifically, group (intervention vs. control) was entered as the independent variable, post-intervention and follow-up outcomes as dependent variables, and the respective baseline scores as covariates. Thus, the reported adjusted Hedge's g_A1 effect sizes represent the baseline-adjusted between-group difference and can be interpreted as the intervention effect.

Changes to the manuscript:

2.3 Feasibility measures (page 9):

To investigate exploratory efficacy on improving EFs and psychosocial variables, ANCOVAs were calculated separately for the post-intervention and the 4-month follow-up outcomes (dependent variables). In each model, group (intervention vs. control) was entered as the independent variable, and the corresponding baseline score as a covariate. This approach tests whether the intervention group showed greater improvement than the control group while adjusting for baseline values. The covariate-adjusted means divided by the the unadjusted post-intervention/4-month follow-up standard deviation can be interpreted as an adjusted Hedge's gA1: large effect sizes (≥ 0.80); moderate effect sizes (≥ 0.50); small effect sizes (≥ 0.20); negligible effect sizes (< 0.20). Accordingly, the reported effect sizes represent the baseline-adjusted between-group difference and can be interpreted as the intervention effect. As no intervention-relevant descriptive variables differed significantly between groups, they were not included as additional covariates in the analysis.

Development and Customization

cCHD-Specific Development: The additional clarification regarding development specific for cCHD is appreciated. However, it would be helpful to elaborate on how

the intervention was customized. Were there any discussions with the coach regarding lived experience? Providing this detail will enhance the manuscript's depth and relevance.

Response: We thank the reviewer for this valuable suggestion. We have added this detail to the description of the coaching to clarify how E-Fit was customized to the lived experience of the children.

Changes to the manuscript:
2.2.1.2 Strategy coaching (page 7):
This session aims to teach them emotion regulation and is either applied to the emotion fear or anger. In this way, coaching sessions incorporate everyday challenges brought forward by children and parents to develop context-specific strategies.

Summary

Overall, the manuscript demonstrates progress and addresses several points raised previously. Addressing the above specific suggestions will further strengthen the paper and its contribution to the field. Thank you for your continued work on this important topic.

Response: We sincerely thank the reviewer for the thoughtful and constructive feedback. We appreciate the recognition of the revisions made and have carefully addressed the additional suggestions to further strengthen the manuscript. We are grateful for the time and expertise invested in improving our work.

Reviewer: 2
Dr. Anne Gallagher, Université de Montréal
Comments to the Author:
The authors have effectively addressed my comments and concerns and should be commended for their efforts. I therefore recommend this manuscript for publication.

Response: We sincerely thank the reviewer for the positive feedback and thoughtful comments throughout the review process. We greatly appreciate the time and expertise invested in evaluating our manuscript.
